# Supplementary material for: Specificity, propagation, and memory of pericentric heterochromatin
Source: Mol Syst Biol. 2014 Aug 18;10(8):746. doi: 10.15252/msb.20145377 (PMC4299515; doi:10.15252/msb.20145377)
Supplement: Supplementary file 1 — Supplementary Information [file msb0010-0746-SD1.docx]

# Supplementary Information

**Specificity, propagation and memory of pericentric heterochromatin**

Katharina Müller-Ott, Fabian Erdel, Anna Matveeva, Jan-Philipp Mallm, Anne Rademacher, Matthias Hahn, Caroline Bauer, Qin Zhang, Sabine Kaltofen, Gunnar Schotta, Thomas Höfer, Karsten Rippe

## Content

[Supplementary Materials and Methods](#_Toc392226425)

- [Plasmid vectors and cell lines](#_Toc392226426)
- [Cell culture, transfections and siRNA knockdown](#_Toc392226427)
- [Immunostaining](#_Toc392226428)
- [Immunoprecipitation, gel electrophoretic analysis and western blots](#_Toc392226429)
- [Real-time quantitative PCR (RT-qPCR)](#_Toc392226430)
- [Analytical ultracentrifugation of recombinant HP1 proteins](#_Toc392226431)
- [Chromatin immunoprecipitation and ChIP-seq data analysis](#_Toc392226432)
- [Confocal laser scanning microscopy (CLSM) and image analysis](#_Toc392226433)
- [Fluorescence recovery after photobleaching (FRAP) and fluorescence (cross-) correlation spectroscopy (FCS/FCCS) data acquisition](#_Toc392226434)
- [Testing the SUV39H long-range methylation activity via a lamina-recruitment assay](#_Toc392226435)
- [Measurements of endogenous protein concentrations and calculation of free protein fraction](#_Toc392226436)
- [Protein-protein interaction analysis via the fluorescent two-hybrid (F2H) assay](#_Toc392226437)

[Supplementary Data Analysis](#_Toc392226438)

- [Fluorescence recovery after photobleaching](#_Toc392226439)
- [Fluorescence correlation spectroscopy](#_Toc392226440)
- [Fluorescence cross-correlation spectroscopy](#_Toc392226441)
- [Estimates of kinetic association rate and equilibrium dissociation constants for SUV39H and HP1](#_Toc392226442)
- [Calculation of H3K9me3 levels](#_Toc392226443)
- [Mathematical modeling of the pericentric heterochromatin network](#_Toc392226444)

[Supplementary Tables](#_Toc392226445) S1-S7

- [Table S1. FCS analysis of HP1α, HP1β, HP1γ and SUV39H1.](#_Toc392226446)
- [Table S2. FRAP analysis of GFP-tagged proteins.](#_Toc392226447)
- [Table S3. Measured and calculated molecular weights and hydrodynamic parameters.](#_Toc392226448)
- [Table S4. Summary of protein-protein associations.](#_Toc392226449)
- [Table S5. Perturbations of PCH features.](#_Toc392226450)
- [Table S6. Cell cycle-dependent mobility of SUV39H1 and HP1α.](#_Toc392226451)
- [Table S7. Parameters used for modeling the PCH network.](#_Toc392226452)

[Supplementary Figures](#_Toc392226453) S1-S5

- [Figure S1. CLSM images of nuclear localization of PCH factors in NIH-3T3 mouse embryonic fibroblasts.](#_Toc392226454)
- [Figure S2. Major repeat transcription measured by RT-qPCR and CLSM analysis of PCH organization in different cell types and knock-out cell lines.](#_Toc392226455)
- [Figure S3. Quantification of protein parameters in living cells by FCS and FRAP](#_Toc392226456)
- [Figure S4. FRAP analysis of JMJD2C, PAX3 and HP1β in *Suv39h* dn and *Suv4-20h* dn cells.](#_Toc392226457)
- [Figure S5. Protein-protein interaction analysis of HP1 and SUV39H1 by FCCS and F2H experiments.](#_Toc392226458)

[Supplementary References](#_Toc392226459)

## Supplementary Materials and Methods

### Plasmid vectors and cell lines

The coding sequences of mouse HP1α,  HP1β  and HP1γ   were cloned into pEGFP-C1 (Clontech) or pTagRFP-C1 (Evrogen) to construct expression vectors for the autofluorescent fusion proteins GFP-HP1α/β/γ or TagRFP-HP1α/β/γ . As an *in vivo* detector for H3K9me3 the chromodomain (CD) of HP1β was inserted into pTagRFP-N. The murine SUV39H1 coding sequence was cloned into either the pEGFP-N1 (Clontech) or the pTagRFP-C1/-N1 (Evrogen) vector (in the following abbreviated as RFP) to generate fluorescently labeled SUV39H1 constructs. The point mutations H320R and H324L (Rea et al, 2000) were introduced into the *Suv39h1* sequence and cloned into the pEGFP-C1 vector. Murine SUV39H2, SUV4-20H1 and SUV4-20H2 were transferred into the vectors pEGFP-N1 or pmCHERRY-N1. Plasmids with GFP-tagged methyl-CpG-binding proteins MBD1, MBD2, MBD3 and MECP2 were kindly provided by Adrian Bird. The pJMJD2C-GFP plasmid was obtained from Barna Fodor (Fodor et al, 2006) and pPAX3/5/7-EGFP and pCAGGS-PAX9-EGFP IRES-Puro were kindly provided by Thomas Jenuwein (Bulut-Karslioglu et al, 2012).

The mRFP1-labeled histone H2A (H2A-RFP) and the GFP-binder (GBP)-LacI construct were described previously (Chung et al, 2011; Jegou et al, 2009) as was the GBP-Lamin B1 plasmid (Rothbauer et al, 2008) and the GFP-HP1α NIH-3T3 cell line (Müller et al, 2009). GFP‑HP1β, GFP-HP1γ and H2A-RFP expressing NIH-3T3 clones were derived after stable transfection with the plasmids described above. The NIH-3T3 SUV39H1-GFP Tet-off cell line was kindly provided by Masato Yonezawa and Thomas Jenuwein. To investigate the interdependencies of protein binding and protein mobility, we compared measurements in wild-type immortalized mouse embryonic fibroblast (iMEF wt) cells and double-null (dn) mutant cells for either the H3K9-specific or the H4K20-specific histone methylases. Both cell lines, iMEF *Suv39h* dn and iMEF *Suv4-20h* dn have been described previously in (Peters et al, 2001) and (Schotta et al, 2008). Protein recruitment experiments were conducted in a human osteosarcoma U2OS cell line with stably integrated *lac* operator (*lac*O) arrays at three different telomeres (clone F6B2) (Jegou et al, 2009).

### Cell culture, transfections and siRNA knockdown

NIH-3T3 cells and derived cell lines as well as the mouse embryonic fibroblast cell lines were cultured in DMEM high glucose medium containing 2 mM L-glutamine (PAA) supplemented with 10 % FCS (PAA) and 1 % penicillin/streptomycin (PAA). For NIH-3T3 SUV39H1-GFP cells 3 µg/ml doxycycline was added to the culture medium to prevent protein overexpression. For induction, the cells were transferred to a new dish without doxycycline two days before the experiment. U2OS F6B2 cells were cultured in regular DMEM medium supplemented with 2 mM L-glutamine, 10 % FCS and 1 % penicillin/streptomycin. For FRAP and FCS experiments, cells were cultured in Labtek chambers (Nunc) and were kept in standard medium using a CO_2_-connected environmental chamber. Alternatively, Leibovitz’s L15 medium (Invitrogen) supplemented with 10 % FCS and 1 % penicillin/streptomycin was used during live experiments. For mobility measurements in mitotic NIH-3T3 SUV39H1-GFP cells, nocodazole (Sigma) was added for 19 h at a final concentration of 100 ng/ml to increase the number of mitotic cells. Inhibition of histone deacetylases in NIH-3T3 or NIH-3T3 SUV39H1-GFP cells was conducted by addition of trichostatin A (TSA) to the medium for up to 5 days at a concentration of 100 ng/ml. Medium and TSA were renewed daily.

Mouse embryonic stem cells (ESCs) from 129P2/Ola mice were cultured in Powerstem ESPro1 medium (Pan-Biotech). Neural progenitor cells (NPCs) were generated from ESCs. The differentiation into NPCs was induced by formation of embryoid bodies in Stempan medium (Pan-Biotech) for 4 d followed by treatment with 5 µM retinoic acid for 4 d. Neuronal embryoid bodies were dissociated and seeded on matrigel (BD Biosciences) in neuronal stem cell medium (Pan-Biotech) for 4 d. Transient (co-)transfection of cells was performed with lipofectamine 2000 (Invitrogen) or turbofect reagent (Thermo Scientific) according to the manufacturers’ protocol. Lipofectamine 2000 was used for the transfection of siRNAs against HP1α, HP1β and HP1γ or a mock siRNA (silencer select siRNAs, Ambion: HP1α (Cbx5) 5’-CAC AGA UUG UGA UAG CAU UTT-3’, HP1β (Cbx1) 5’-GGA UUG CCC UGA CCU UAU UTT-3’, HP1γ (Cbx3) 5’-CAC AGA UGC UGA UAA UAC UTT-3’, negative control #2). A mix of 60-120 nM of each siRNA was added to the cells. Cells were fixed and immunostained for microscopy analysis after 48 h. For transfection of human cells (U2OS F6B2) the effectene transfection reagent (Qiagen) was used according to the manufacturers’ protocol and cells were fixed 24 h after transfection.

### Immunostaining

For immunofluorescence imaging, cells were fixed on glass coverslips with 4 % paraformaldehyde, permeabilized for 3-5 min with 0.5 % triton X-100 in PBS or CSK-buffer (100 mM NaCl, 300 mM sucrose, 3 mM MgCl_2_ and 10 mM PIPES pH 6.8, 0.5 % triton X-100) and blocked with 10 % goat serum in PBS or alternatively with 3 % BSA in PBS. Immunostaining of fixed cells was conducted with primary anti-H3K9me3 (Millipore; Abcam ab8898), anti-HP1α (Euromedex, 2HP-1H5-AS), anti-HP1β (Euromedex, 1MOD-1A9-AS), anti-HP1γ (Euromedex, 2MOD-1G6-AS), anti-H4K20me3 (Abcam, ab9053) or anti-5-methylcytosine (Calbiochem, 16233D3) antibodies, and subsequent visualization was performed with a secondary goat anti-rabbit/mouse Alexa 568 antibody or anti-rabbit/mouse Alexa 633 antibody (Invitrogen). Cells were mounted with Prolong Gold antifade reagent (Invitrogen) containing 4’,6-diamidino-2’-phenylindole (DAPI) for the staining of chromatin/DNA. For immunofluorescent labeling of 5-methylcytosine, fixed cells were denatured with 4 N HCl and 1 % triton X-100 in water for 15 min. After three washing steps with PBS supplemented with 0.002 % NP-40, blocking and antibody labeling was conducted as described above.

### Immunoprecipitation, gel electrophoretic analysis and western blots

NIH-3T3 cells stably expressing GFP-HP1β and GFP-HP1γ were lysed in an ice-cold buffer containing 10 mM Tris-HCl pH 7.5, 150 mM NaCl, 0.5 mM EDTA, 0.1 % NP-40, 1 mM PMSF, and a complete protease inhibitor cocktail (Roche) for 30 min. For the analysis of the lowly abundant SUV39H1, NIH-3T3 SUV39H1-GFP cells were induced for 48 h and lysed with a buffer containing 50 mM Tris-HCl, pH 7.5, 200 mM NaCl, 5 mM EDTA, 1 % triton X-100 supplemented with PMSF and protease inhibitor cocktail. Subsequently, endogenous and GFP-tagged SUV39H1 were immunoprecipitated to concentrate the protein by addition of 2.5 µg of anti-SUV39H1 antibody (Abcam ab38637) bound to protein G magnetic beads (Roth) at 4 °C overnight and crosslinked to the beads with DMP (dimethyl pimelimidate dihydrochloride). Antigen was eluted from the beads with 0.1 M glycine pH 2.5, and samples were analyzed by SDS-PAGE. Further analysis was either by Coomassie staining for immunoprecipitated SUV39H1 or by western blotting after transfer to a nitrocellulose membrane (Whatman) with the following primary antibodies: anti-HP1β (Abcam, ab10478, 1 : 500), anti-HP1γ (Euromedex, 2MOD-1G6-AS, 1 : 1000) and anti-SUV39H1 (Cell Signaling, 1 : 800) and HRP-conjugated anti-mouse IgG or anti-rabbit IgG secondary antibodies (Cell Signaling, 1:1000).

For quantification a chemiluminescent ECL reagent (1 ml 0.1 M Tris-HCl, pH 7.5 supplemented with 0.25 mg luminol, 0.3 μl H2O2, 100 μl DMSO, 0.11 mg para-hydroxycoumarin acid) was used and the signal was recorded using an INTAS ChemoCam Imager equipped with a 16-bit camera. The protein ratio was quantified with an ImageJ-based intensity analysis of the protein bands from western blots (HP1α/β/ γ).

### Real-time quantitative PCR (RT-qPCR)

Total RNA was extracted from three different samples (ESCs, iMEF wt and iMEF *Suv39h* dn cells) with the RNeasy Mini Kit (Qiagen) including an additional DNase digestion step with 50 U DNase I at 37 °C for 1 h. First-strand cDNA was transcribed from 2 µg RNA using 8.3 µM random hexamer primers (Thermo Scientific), 0.83 mM dNTPs and 10 U/µl SuperScript II reverse transcriptase (Invitrogen). Residual RNA fragments were digested by addition of 10 U RNase H. 1 % of the cDNA was used as input for the RT-qPCR conducted with a Light Cycler 480 SybrGreen I Master mix (Roche) and 0.5 µM of specific primers with a StepOne Real Time PCR-System (Applied Biosystems). For the RT-qPCR standard curve total RNA (20 µg/µl) of ESCs was used and the resulting cDNA was applied from 0.2-60 ng/µl. The following primer sequences were used: minor forward, 5’-AAT GAT AAA AAC CAC ACT GTA GAA CAT-3‘, minor reverse, 5‘-ATG TTT CTC ATT GTA ACT CAT TGA TAT AC-3‘, major forward, 5‘-TGG CGA GAA AAC TGA AAA TCA CG-3‘, major reverse, 5‘-TCT TGC CAT ATT CCA CGT CCT AC-3‘, GAPDH forward, 5‘-TAT GTC GTG GAG TCT ACT GG-3‘ and GAPDH reverse 5‘-ACA CCC ATC ACA AAC ATG GG-3‘.

### Analytical ultracentrifugation of recombinant HP1 proteins

Analytical ultracentrifugation (AUC) experiments of full-length HP1β  and its isolated chromodomain (CD) and chromoshadow domain (CSD) in approximately physiological buffer was characterized by analytical ultracentrifugation (AUC) with a Beckman Instruments Optima XL-A with absorbance optics at 20 °C as described previously (Fejes Tóth et al, 2005; Kepert et al, 2003). The construct for mouse full-length HP1β as well as its CD (residues 10-80) and CSD (residues 104-185) were kindly provided by Natasha Murzina. Proteins were expressed in *E.coli* BL21 pLysE cells from pET 16d (full-length protein and CSD) or pET 24d (CD) vectors with an N-terminal His-tag. Cleared cell lysates were incubated with 0.5 ml of Ni-NTA resin (Qiagen) per liter of bacterial culture and allowed to bind for at least one hour. After washing the resin with increasing concentrations of imidazole (0-20 mM), the proteins of interest were eluted with 200 mM imidazole in 20 mM Tris-HCl, pH 7.5 supplemented with 100 mM KCl. Protein fractions were pooled and further purified by size-exclusion chromatography.

AUC experiments with full length HP1β and its CD and CSD domains were conducted in a buffer containing 10 mM Tris-HCl, pH 7.5 and 100 mM KCl in the range from 1-30 µM monomer concentration. Absorbance was recorded at 280 nm or 230 nm. Partial specific volumes as listed in Supplementary Table S3 were calculated with the program Sednterp V1.05 by J. Philo, D. Hayes and T. Laue (Laue et al, 1992). The same program was used to compute buffer densities and viscosities. The sedimentation velocity runs were conducted at 48 000 rpm with 0.003 cm spacing in the continuous scan mode. Data were analyzed with the programs Sedfit 9.4 (Schuck, 2003) and dcdt+, version 1.16 (Philo, 2000). Sedimentation equilibrium experiments were conducted at 10 000, 15 000 and 20 000 rpm using a spacing of 0.001 cm and 10 replicates in the step-wise scan mode. Data were analyzed with the Ultrascan software, version 8.0 (Demeler, 2005). Equilibrium data sets from three different protein loading concentrations at three speeds were used for global curve fitting analysis to a one-component model. To determine the stability of the HP1β dimer the AUC analysis was extended to lower protein concentrations by absorbance detection of the concentration profile at 230 nm. The extinction coefficient at this wavelength was about 6 times higher than at 280 nm with a value of ∑230 = 183 000 M^-1^ cm^-1^ so that measurements down to 1 μM could be conducted.

### Chromatin immunoprecipitation and ChIP-seq data analysis

ChIP-seq experiments were conducted as described previously (Teif et al, 2012). For each sample, 1 x 10^6^ cells were cross-linked with 1 % formaldehyde for 10 minutes and chromatin was rendered accessible using a swelling buffer (25 mM Hepes, pH 7.8, 1 mM MgCl_2_, 10 mM KCl, 0.1% NP-40, 1 mM DTT). Chromatin was sheared to 220 bp fragments for HP1 and SUV39H and to 150 bp for histone modifications by sonification. After IgG preclearance, the sheared chromatin was incubated with 4 µg of antibody directed against H3K9me3 (Abcam, ab8898), H3K36me3 (Abcam, ab9050), SUV39H1 (Abcam, ab12405), SUV39H2 (Abcam, ab5264) or HP1β (Euromedex, 1MOD-1A9-AS) and protein G magnetic beads overnight. After washes with sonication buffer (10 mM Tris-HCl, pH 8.0, 200 mM NaCl, 1 mM EDTA, 0.5% N-lauroylsarcosine, 0.1% Na-deoxycholate), high-salt-buffer (50 mM Hepes pH 7.9, 500 mM NaCl, 1mM EDTA, 1% triton X-100, 0.1% Na-deoxycholate, 0.1% SDS), lithium buffer (20 mM Tris-HCl pH 8.0, 1 mM EDTA, 250 mM LiCl, 0.5 % NP-40, 0.5 % Na-deoxycholate) and 10 mM Tris-HCl, chromatin was eluted from magnetic beads and the crosslink was reversed over night at 65 °C. After RNase A and proteinase K digestion, DNA was purified and cloned into a barcoded sequencing library for the Illumina HiSeq2000 sequencing platform (single reads of 50 bp length).

For ChIP-seq data analysis the reads were uniquely mapped with Bowtie without mismatches to the mm9 mouse genome. From 42 intergenic/intronic major gamma satellite (GSAT) repeats annotated in the Repeatmasker software only 16 were uniquely mappable. The read density at these 16 genomic positions was normalized to the total read number and the enrichment was calculated by dividing the read density of ChIP by input samples. To calculate the coverage over the pericentric major satellite repeat consensus sequence (GGA CCT GGA ATA TGG CGA GAA AAC TGA AAA TCA CGG AAA ATG AGA AAT ACA CAC TTT AGG ACG TGA AAT ATG GCG AGG AAA ACT GAA AAA GGT GGA AAA TTT AGA AAT GTC CAC TGT AGG ACG TGG AAT ATG GCA AGA AAA CTG AAA ATC ATG GAA AAT GAG AAA CAT CCA CTT GAC GAC TTG AAA AAT GAC GAA ATC ACT AAA AAA CGT GAA AAA TGA GAA ATG CAC ACT GAA, (Lehnertz et al, 2003)), the repeat sequence was circularized to allow all reads to map to two adjacent repeats. Normalization and enrichment was then calculated as described above. Errors were calculated from two independent ChIP-seq experiments. ChIP-seq data produced in the current study have been deposited in the GEO database (accession number GSE58555).

### Confocal laser scanning microscopy (CLSM) and image analysis

For standard confocal imaging a Leica TCS SP5 confocal laser scanning microscope was used as described previously (Müller et al, 2009). 3D image stacks were taken with 0.4 µm sections. For quantitative analysis of protein enrichment and chromatin density in pericentric heterochromatin relative to euchromatin, images were acquired with a Zeiss LSM 710 confocal microscope equipped with a UV-diode and an Argon multi-line laser, a 63x/1.4 NA oil DIC III objective and high sensitivity avalanche photodiode detectors (APD-imaging). Image analysis was conducted with the ImageJ software. Protein enrichment in PCH as defined by DAPI-dense foci was calculated by measuring the mean intensity values in the protein or histone modification channels (either GFP/RFP-tagged or immunofluorescently labeled) in those foci relative to the average intensity measured outside of these regions after correction for background noise. The comparative analysis of H3K9me3 levels in iMEF wt and iMEF *Suv39h* dn cells was conducted by quantitating immunostaining signals in PCH and euchromatin for both cell lines on the same slide. Measured signals were converted to H3K9me3 levels by using the nuclear trimethylation level of 28 ± 2 % in wild-type NIH-3T3 cells determined by mass spectrometry (Fodor et al, 2006) as well as the relative genomic fraction of 3.6 % for major satellite repeats (Waterston et al, 2002). A similar analysis was conducted for the HP1 triple knockdown (TKD) experiments. After maximum intensity projection, protein expression and methylation levels were analyzed as described above. To estimate knockdown levels, the HP1 intensity of HP1 siRNA-transfected cells was compared to that of mock siRNA-transfected cells and normalized to this value.

2D confocal images of JMJD2C-GFP expressing cells were segmented using binary masks for the nucleus and PCH foci (derived from DAPI images) to yield the per-nucleus-expression of JMJD2C-GFP and the H3K9me3 enrichment in PCH foci. The H3K9me3 immunofluorescence signal was normalized to the DAPI signal and averaged over all pixels belonging to an individual PCH focus. The JMJD2C-GFP expression level was given as the mean JMJD2C-GFP signal of all pixels belonging to an individual nucleus. With these parameters, the H3K9me3 enrichment versus the corresponding JMJD2C-GFP expression level was determined and discretized into 20 equally sized bins of JMJD2C-GFP levels.

Cell cycle states were assigned based on a combination of different markers that included the punctuate PCNA distribution observed during S phase, the localization of HP1 to PCH, which is indicative of G1 or S phase, and the phosphorylation of histone H3 at serine 10, which occurs during G2/M phase. While PCNA and H3S10p were used for fixed samples, the co-transfection of PCNA and/or HP1 was used to identify cell cycle phases in living cells.

### Fluorescence recovery after photobleaching (FRAP) and fluorescence (cross-) correlation spectroscopy (FCS/FCCS) data acquisition

For FRAP experiments, images were acquired on a Leica TCS SP5 confocal microscope at 128 x 128 pixels and 1400 Hz scanning speed at a time resolution of 115 ms per image. Typically, 25-50 images were acquired before bleaching twice (i.e. 230 ms) at 100 % laser power. Subsequently, images were acquired for 4 min. The FRAP data analysis is described in the Supplementary Data Analysis section.

FCS and FCCS experiments were conducted with a Zeiss LSM 710 microscope equipped with a 63x/NA 1.2 water immersion objective with correction collar and a ConfoCor3 unit using the program ZEN (Zeiss). GFP and RFP excitation was done with a 488 nm Argon laser line and a 561 nm laser line of a diode-pumped solid-state laser. Emitted signals were recorded through a BP 505-540 IR and a LP 580 filter to separate both channels.

The calibration of the focal volume dimensions was done with an aqueous solution of TetraSpeck beads (Life Technologies). For F(C)CS measurements, confocal images were taken to determine the region for the fluctuation measurements in the cytoplasm, euchromatin and heterochromatin. Concentration fluctuations of fluorescently labeled molecules were recorded for 60 s and the intensity signal was subjected to a time correlation analysis to obtain auto- or cross-correlation functions. The data analysis is described below.

### SUV39H long-range methylation activity upon lamina-recruitment

To test the ability of SUV39H1 to methylate nucleosomes at some spatial distance, we tethered GFP-SUV39H1 or inactive SUV39H1-H324L-GFP (negative control) to the nuclear lamina via a GFP binding protein (GBP) fused to Lamin B1 (GBP-Lamin B1). H3K9me3 levels in iMEF *Suv39h* dn cells were detected by a red fluorescent construct of the HP1 chromodomain (CD-RFP) after transfection with GBP-Lamin B1 and GFP-SUV39H1 or SUV39H1-H324L-GFP. H3K9me3 enrichment at the nuclear lamina was determined by averaging the fluorescence intensity profiles of GFP-tagged SUV39H and CD-RFP from the nuclear boundary towards the nucleus center over the entire nuclear lamina. By fitting these profiles for GFP-SUV39H1 and CD-RFP with an exponential function, the full width at half-maximum was determined, which describes the spatial extension of the newly formed H3K9me3 modification domains.

### Measurements of endogenous protein concentrations and calculation of free protein fraction

The concentrations of GFP-tagged SUV39H1, HP1α/β/γ and SUV4-20H proteins in eu- and heterochromatin were measured by FCS in cell lines that stably expressed these proteins as described previously (Müller et al, 2009) (Supplementary Table S1). The ratios between endogenous and GFP-tagged proteins were determined by quantitative analysis of immunoprecipitated protein or cell lysate on Coomassie-stained gels and by western blotting (Supplementary Fig S3 B, C, Supplementary Table S1, Table 1). The corresponding concentrations for SUV4-20H were derived from FCS measurements in ESCs with a GFP knock-in at the C-terminus of the endogenous genes. Since SUV4-20H concentrations do not change significantly during differentiation of ESCs (Efroni et al, 2008), these concentrations were used here also for fibroblasts. The concentrations of SUV39H2, MECP2 and MBD1 were estimated from RNA expression levels determined by RNA-seq in MEFs. In these cells a SUV39H1 to SUV39H2 transcript ratio of 3.4 : 1 was determined, the transcript ratio of SUV39H1 to MECP2 was 0.34 : 1 and that of SUV39H1 to MBD1 was 1.36 : 1.

Based on the endogenous protein concentration the free protein concentration of HP1 and SUV39H was calculated as follows: The diffusion coefficient measured with FCS reflects both freely diffusive and transiently bound proteins. The (effective) diffusion coefficient measured in euchromatin was smaller due to chromatin-binding interactions compared to the diffusion coefficient of free protein measured in the cytoplasm. The ratio between both values is $D_{\text{FCS,Eu}}/D_{\text{FCS,Cyto}}=1/\left(1+k_{\text{on}}^{\text{*}}/k_{\text{off}}\right)$ (Sprague et al, 2004) with pseudo-association rate *k**_on_ and dissociation rate *k*_off_. Accordingly, the free protein fraction *f*_free_ was calculated from $f_{\text{free}}=k_{\text{off}}/\left(k_{\text{on}}^{\text{*}}+k_{\text{off}}\right)=1/\left(1+k_{\text{on}}^{\text{*}}/k_{\text{off}}\right)$. The free protein concentration *c*_free_ was then obtained by multiplying *f*_free_ with the concentration of the total mobile protein pool that contributes to the effective diffusion coefficient measured by FCS. In euchromatin this protein fraction was represented by binding site classes I and II for HP1 and by class I for SUV39H (see Table 1 and Supplementary Data Analysis “Fluorescence recovery after photobleaching” below).

### Protein-protein interaction analysis via the fluorescent two-hybrid (F2H) assay

The interaction of HP1 dimers with SUV39H could not be measured reliably by FCCS due to the large difference between SUV39H1 and HP1 concentrations that leads to a high background from the HP1 signal. Furthermore, SUV39H1 proteins were bound to chromatin with high affinity and thus were subject to fluorescence photobleaching, rendering them unsuitable for FCCS experiments. Accordingly, we applied the fluorescent two-hybrid assay (F2H) to study interactions of HP1, SUV39H1, MECP2 and MBD1 in living cells. The GFP-labeled protein was tethered to integration sites of *lac* operator (*lac*O) repeats referred to as a *lac*O-array in a U2OS cell line (F6B2) via a bacterial LacI repressor fused to a GFP-binding protein (LacI-GBP) as described previously (Chung et al, 2011; Rothbauer et al, 2006; Zolghadr et al, 2008). For the interaction analysis 3D image stacks spaced 0.25 µm along the z-axis were recorded. Maximum intensity projections of these stacks were then analyzed for recruitment of the GFP-tagged protein to the *lac*O-arrays and colocalization of the RFP-labeled interaction partner at these foci (Fig 2C, Supplementary Fig S5B). Spots were counted as positive for colocalization if the background-corrected RFP-signal at the *lac*O-arrays was at least 1.7-fold above the average nuclear intensity level. This type of analysis was conducted for the dimerization of SUV39H1 (SUV39H1-GFP, SUV39H1-RFP) and to evaluate the interactions of MBD-proteins (MBD1 and MECP2) with HP1 or SUV39H.

## Supplementary Data Analysis

### Fluorescence recovery after photobleaching

FRAP analysis was carried out with the software FREDIS (Müller et al, 2009) by fitting the time evolution of the intensity integrated over an effective 1.9 µm bleach spot (1.5 µm bleaching diameter plus 0.4 µm broadening due to fast diffusion processes occurring during the bleach) either to a diffusion model, to a reaction model or to a reaction-diffusion model that incorporates both diffusion and binding processes based on the theoretical framework by (Sprague et al, 2004). To select the best fit model for each FRAP curve, the F-ratio was calculated that accounts for the relative change in the sum of residuals and the relative change in degrees of freedom of two different models. In general, the reaction-diffusion model gave the best results except for HP1 and SUV4-20H in euchromatin that were described best by an effective diffusion model. This model is appropriate if either no binding sites or binding sites with very high dissociation rates are present. The latter results in an effective diffusion coefficient *D*_eff_ and a parameter *f*_im_ that describes the fraction of proteins immobilized during the measurement. The reaction-diffusion model describes the recovery in the presence of a high affinity binding site characterized by the dissociation constant *k*_off_, the equilibrium constant *K**_eq_ and the fraction of transiently bound molecules. The diffusion coefficients obtained in the nucleus were significantly smaller than the free diffusion coefficient measured by FCS (see below) in the cytoplasm. Since free diffusion is expected to be similar in the cytoplasm and the nucleus (Pack et al, 2006), the reduced diffusion coefficient extracted from both diffusion and reaction-diffusion model can be explained by transient chromatin interaction that cannot be resolved and thus reflects low affinity binding referred to here as class I binding sites. Due to the 115 ms time resolution of a single image frame, the residence time at class I sites can be estimated to be ≤ 0.5 s. The immobile fraction extracted from both fit models depicts the highest affinity binding sites and was identified as binding class IV. Binding classes II and III were extracted from reaction-diffusion fits. In addition, we compared mobility parameters in euchromatin and PCH for a given protein to determine the minimal number of binding site classes that described our experimental data in a consistent manner. For example, we observed that the kinetic off-rate of SUV39H1 in PCH from the reaction-diffusion fit was more than three-fold lower than in euchromatin. Accordingly, we interpreted the heterochromatic off-rate as an average value for the interaction with both euchromatin and PCH binding sites and recalculated the data with a reaction-diffusion model with two binding states. Based on the assumption that the bound fraction and the residence time (*k*_off_^-1^) are the weighted averages of the bound fractions/residence times of class II and class III, the kinetic rate constants for class II and class III are given by

$\begin{matrix} k_{\text{off.III}}=\frac{k_{\text{off,eff}}k_{\text{off.II}}\left(k_{\text{on,eff}}k_{\text{off,II}}-k_{\text{on,II}}k_{\text{off,eff}}\right)}{k_{\text{on,eff}}k_{\text{off,II}}^{2}-k_{\text{on,II}}k_{\text{off,eff}}^{2}} \\ k_{\text{on,III}}=\frac{\left(k_{\text{on,eff}}k_{\text{off,II}}-k_{\text{on,II}}k_{\text{off,eff}}\right)^{2}}{k_{\text{on,eff}}k_{\text{off,II}}^{2}-k_{\text{on,II}}k_{\text{off,eff}}^{2}} \end{matrix}$ (1)

Since class II was assigned to the binding reaction in euchromatin, the rates $k_{\text{on,III}}$ and $k_{\text{off,III}}$ were obtained based on the euchromatic values $k_{\text{on,II}}$/$k_{\text{off,II}}$ and the average heterochromatic values $k_{\text{on,eff}}$/$k_{\text{off,eff}}$.

For HP1 in euchromatin the diffusion model was applied but resulted in a very small effective diffusion coefficient. Thus, we concluded that the low affinity binding sites identified in PCH with a reaction-diffusion model fit were also present in euchromatin but at a lower concentration. Accordingly, we refitted the recovery curves with a reaction-diffusion model and fixed *k*_off_ at the value that was measured in heterochromatin.

### Fluorescence correlation spectroscopy

Data analysis including calculation of the autocorrelation function (ACF) was conducted with a half-automated self-written FCS analysis software tool termed Spatiotemporal Correlation Suite (STCor). STCor reads the Zeiss fluctuation raw data files (*.raw) and calculates the autocorrelation function according to the formalism described in our previous work (Müller et al, 2009). The ACFs were fit to an anomalous diffusion model with one (cytoplasm) or two (eu-/heterochromatin) components. Protein concentrations were calculated in STCor from the particle number within the focal volume obtained from the fit. The (effective) diffusion coefficients were calculated from the measured diffusion times compared to the diffusion time and the diffusion coefficient of Tetraspeck beads (Muller et al, 2008). The anomaly parameter characterizing the nonlinear time dependence of the mean squared particle displacement was obtained for both fractions and typically was > 1 for the second fraction. Since this is indicative of confined diffusion behavior and typically observed for proteins that interact transiently with chromatin in FCS measurements, the second component reflects chromatin translocations that can be observed as slow intensity fluctuations of chromatin-bound fluorescently labeled molecules (Erdel et al, 2011; Erdel et al, 2010; Müller et al, 2009).

### Fluorescence cross-correlation spectroscopy

The interaction of GFP- and RFP-tagged proteins in NIH-3T3 cells was measured by fluorescence cross-correlation spectroscopy with a Zeiss LSM 710 ConfoCor3 as described above. The presence of endogenous unlabeled protein as well as complexes containing only particles labeled with one color (e.g. (HP1)_2_, (GFP-HP1)_2_ or (RFP-HP1)_2_) reduces the FCCS signal. Thus, the fraction of the total amount of proteins incorporated into a multimeric complex was calculated from the amplitudes of the auto- (ACF) and cross-correlation functions (XCF). For this analysis, spectral crosstalk was corrected according to

$\begin{matrix} G_{\text{green}}(\tau)=G_{\text{green}}^{\text{*}}(\tau) \\ G_{\text{red}}(\tau)=\frac{1}{\left(1-\gamma\right)^{2}}\left(G_{\text{red}}^{\text{*}}(\tau)-2\gamma G_{\text{cross}}^{\text{*}}(\tau)+\gamma^{2}G_{\text{green}}^{\text{*}}(\tau)\right) \\ G_{\text{cross}}(\tau)=\frac{1}{1-\gamma}\left(G_{\text{cross}}^{\text{*}}(\tau)-\gamma G_{\text{green}}^{\text{*}}(\tau)\right) \end{matrix}$ (2)

Here, *G*_i_* are the measured correlation functions including crosstalk and *G*_i_ are the corrected correlation functions. Further, $\gamma=\kappa\left\langle I_{\text{g}}\right\rangle\left\langle I_{\text{r}}\right\rangle^{-1}$ with $\kappa$ being the brightness of the green fluorophore (GFP) in the red channel divided by its brightness in the green channel, and $\left\langle I_{\text{g}}\right\rangle$ and $\left\langle I_{r}\right\rangle$ representing the average intensity in the green and red channel, respectively. A similar crosstalk correction was reported recently by (Bacia et al, 2012). To measure the brightness of GFP in the red channel, cells were transfected with inert GFP protein and a standard measurement was conducted with both lasers and detection in both the red and the green channel. Data fitting of the crosstalk corrected functions was done as described previously (Erdel et al, 2010; Müller et al, 2009) using the STCor software.

The degree of cross-correlation, *ratioG*, i.e. the fraction of double-labeled molecules normalized to the number of molecules of each ‘color’ was calculated according to

$ratio G=\frac{G_{\text{cross}}(0)}{G_{\text{green}}(0)\cdot G_{\text{red}}(0)}$. (3)

Here, *G*_cross_(0) denotes the amplitude of the cross-correlation function and *G*_green_(0) and *G*_red_(0) denote the amplitudes of the autocorrelation function in the green and the red channel at lag time τ = 0, respectively. The fraction of monomeric protein $\Theta$ that is incorporated into a homodimeric complex is related to *ratio G* via

$ratioG=\Theta\sqrt{\frac{\lambda_{\text{g}}\lambda_{\text{r}}}{\left(1+\lambda_{\text{g}}\Theta\right)\left(1+\lambda_{\text{r}}\Theta\right)}}$ (4)

Here, $\lambda_{\text{g}}$ and $\lambda_{\text{r}}$ are the label degrees for the green and red species, respectively. This result is obtained by using the definition of the correlation function for partially labeled pools (Erdel et al, 2010; Weidemann et al, 2002). This relation can be inverted to obtain the fraction of proteins in dimeric complexes based on the label degrees and *ratio G*:

$\Theta=\sqrt{\frac{\left(\lambda_{\text{g}}+\lambda_{\text{r}}\right)^{2}ratioG^{4}}{4\lambda_{\text{g}}^{2}\lambda_{\text{r}}^{2}\left(ratioG^{2}-1\right)^{2}}-\frac{ratioG^{2}}{\lambda_{\text{g}}\lambda_{\text{r}}\left(ratioG^{2}-1\right)}}-\frac{\left(\lambda_{\text{g}}+\lambda_{\text{r}}\right)ratioG^{2}}{2\lambda_{\text{g}}\lambda_{\text{r}}\left(ratioG^{2}-1\right)}$ (5)

### Estimates of kinetic association rate and equilibrium dissociation constants for SUV39H and HP1

Dissociation constants for the independent binding of SUV39H and HP1 can be estimated from FRAP data as follows: The pseudo-affinity $K_{\text{eq}}^{\text{*}}=k_{\text{on}}^{\text{*}}/k_{\text{off}}=k_{\text{on}}c_{\text{s}}/k_{\text{off}}\approx5.1$ for HP1 was derived from the FRAP fit curves (Supplementary Table S2). Since binding to trimethylated H3K9 residues is the main chromatin interaction of HP1 in the nucleus (Müller et al, 2009), the substrate concentration *c*_s_ in the above equation can be approximated with the concentration of trimethylated nucleosomes, which equals roughly 100 µM in heterochromatin (Fodor et al, 2006; Wachsmuth et al, 2008). Based on these values the association rate for HP1 was calculated according to $k_{\text{on}}=K_{\text{eq,HP1}}^{\text{*}} k_{\text{off,HP1}}/c_{\text{s}}\approx1.8\cdot10^{4}M^{-1}s^{-1}$. This corresponds well to association rates measured for other nuclear proteins (Phillip et al, 2012). The dissociation constant for HP1 is $K_{\text{d,HP1}}=k_{\text{off,HP1}}/k_{\text{on}}=c_{\text{s}}/K_{\text{eq,HP1}}^{\text{*}}\approx20\mu M$. This rather low affinity reflects the transient binding behavior observed for the majority of HP1 molecules. As shown by FCS measurements in the cytosol, the free diffusion coefficients of HP1 and SUV39H are similar (Supplementary Table S1), suggesting that their (diffusion-limited) association rate can be approximated with the same value (Berg & von Hippel, 1985). Accordingly, the dissociation constant for transient SUV39H binding was estimated from the dissociation rate determined by FRAP in heterochromatin (Supplementary Table S2) to be $K_{\text{d,SUV39H}}=k_{\text{off,SUV39H}}/k_{\text{on}}\approx3.5\mu M$. Thus, individual HP1 and SUV39H molecules bind relatively weakly to chromatin. However, due to their interaction, the affinity of the HP1-SUV39H complex to chromatin is much higher. Assuming that the individual chromatin binding contributions in the HP1-SUV39H complex are additive with binding energies ∆*G*_HP1_ for HP1 and ∆*G*_SUV39H_ for SUV39H1 one obtains Δ*G*_HP1-SUV39H_ = Δ*G*_HP1_ + Δ*G*_SUV39_, which corresponds to $K_{\text{d,HP1-SUV39H}}=\exp\left(-\left(\Delta G_{\text{HP1}}+\Delta G_{\text{SUV39H}}\right)/RT\right)\approx0.07nM$ for the HP1-SUV39H complex (with ∆*G*_HP1_ ≈ 27 kJ/mol and ∆*G*_SUV39H_ ≈ 31 kJ/mol). Since HP1 and SUV39H interact with each other in a dynamic equilibrium, this *K*_d_ value can be considered to represent a lower limit.

### Calculation of H3K9me3 levels

To estimate the relative methylation rates in eu- and heterochromatin we used a simplified description in which the nucleosomes can adopt an unmodified state (U), a methylated state (M) and also an ‘anti-modified’ state, i.e. carrying the opposing acetylation modification (A):

This model in steady-state is described by the following equations:

$\begin{matrix} \frac{\partial M}{\partial t}=U\cdot k_{\text{m}}-M\cdot k_{-\text{m}}=0 \\ \frac{\partial A}{\partial t}=U\cdot k_{\text{a}}-A\cdot k_{-\text{a}}=0 \\ \frac{\partial U}{\partial t}=M\cdot k_{-\text{m}}+A\cdot k_{-\text{a}}-U\cdot\left(k_{\text{a}}+k_{\text{m}}\right)=0 \end{matrix}$ (6)

With the normalization $M+A+U=1$, the steady-state methylation level for this system is given by

$M(K,r)=\frac{r}{1+K+r}$ (7)

with the rate ratios $K=k_{\text{a}}/k_{\text{-a}}$ and $r=k_{\text{m}}/k_{\text{-m}}$. Using the relative H3K9me3 levels determined by immunostaining (Supplementary Fig S2B, Fig 3C), the following relations for the rate constants are obtained:

$\begin{matrix} r_{\text{Eu,wt}}=\left(\frac{k_{\text{m}}}{k_{\text{-m}}}\right)_{\text{Eu,wt}}=\frac{M\left(1+K\right)}{1-M} r_{\text{Eu,KO}}=\left(\frac{k_{\text{m}}}{k_{\text{-m}}}\right)_{\text{Eu,KO}}=\frac{M c_{2}\left(1+K\right)}{1-M c_{2}} \\ r_{\text{Het,wt}}=\left(\frac{k_{\text{m}}}{k_{\text{-m}}}\right)_{\text{PCH,wt}}=\frac{M c_{1}\left(1+K\right)}{1-M c_{1}} r_{\text{PCH,KO}}=\left(\frac{k_{\text{m}}}{k_{\text{-m}}}\right)_{\text{PCH,KO}}=\frac{M c_{3}\left(1+K\right)}{1-M c_{3}} \end{matrix}$ (8)

Here, *k*_m_ and *k*_-m_ are the methylation and demethylation rates for eu-/heterochromatin in the respective cell line, *M* = 28 % is the methylation level in wild-type euchromatin (Fodor et al, 2006), and *c*_1_ - *c*_3_ are the methylation levels of heterochromatin in wild-type cells (*c*_1_ = 1.4), euchromatin in *Suv39h* dn cells (0.75 < *c*_2_ < 1) and heterochromatin in *Suv39h* dn cells (*c*_3_ = 0.5) relative to the methylation level of wild-type euchromatin (*M*), respectively. Since both SETDB1/G9A and SUV39H are known to catalyze H3K9me3, the methylation rate can be split up into the contributions from these two enzymes: *k*_m,x_ = *k*_SETDB1/G9A,x_ + *k*_SUV39H,x_. Since both methylases are differentially regulated in eu-/heterochromatin, individual rate constants were assigned for the different chromatin types. In *Suv39h* dn cells, the SUV39H rates are zero, i.e. *k*_SUV39H,Eu,KO_ = *k*_SUV39H,Het,KO_ = 0. Thus, the rates of the two enzymes are related to the expression in equation (8) by

$$\begin{matrix} \left(\frac{k_{\text{SETDB1/G9A}}}{k_{\text{-m}}}\right)_{\text{Eu}}=r_{\text{Eu,KO}}=\frac{M c_{2}\left(1+K\right)}{1-M c_{2}} & \left(\frac{k_{\text{SUV39H}}}{k_{\text{-m}}}\right)_{\text{Eu}}=r_{\text{Eu,wt}}-r_{\text{Eu,KO}}=\frac{M\left(1+K\right)\left(1-c_{2}\right)}{\left(1-M\right)\left(1-M c_{2}\right)} \\ \left(\frac{k_{\text{SETDB1/G9A}}}{k_{\text{-m}}}\right)_{\text{PCH}}=r_{\text{PCH,KO}}=\frac{M c_{3}\left(1+K\right)}{1-M c_{3}} & \left(\frac{k_{\text{SUV39H}}}{k_{\text{-m}}}\right)_{\text{PCH}}=r_{\text{PCH,wt}}-r_{\text{PCH,KO}}=\frac{M\left(1+K\right)\left(c_{1}-c_{3}\right)}{\left(1-M c_{1}\right)\left(1-M c_{3}\right)} \end{matrix}$$

(9)

The steady-state methylation levels observed by immunostaining in Fig 3C can be explained by the model given above with the values for the relative methylation rates in equations (8) and (9). For simplicity, the acetylation state was not taken into account, i.e. *k*_a_ = 0. This seems to be justified since less than 13% of the nucleosomes are acetylated (Fodor et al, 2006). The specificity of both methylases is reflected by the ratio of their rate constants in euchromatin and PCH, which is independent of *K* and amounts to

$\frac{k_{\text{SUV39H,PCH}}}{k_{\text{SUV39H,Eu}}}=\frac{\left(1-M c_{2}\right)\left(1-M\right)\left(c_{1}-c_{3}\right)}{\left(1-c_{2}\right)\left(1-M c_{1}\right)\left(1-M c_{3}\right)}>3.8, \frac{k_{\text{SETDB1/G9A,Eu}}}{k_{\text{SETDB1/G9A,PCH}}}=\frac{c_{2}\left(1-M c_{3}\right)}{c_{3}\left(1-M c_{2}\right)}>1.8$ (10)

As expected, SUV39H has a preference for PCH and SETDB1/G9A for euchromatin, respectively. The methylation rate of SUV39H is at least 4-fold increased in PCH and the methylation rate of G9A/SETDB1 is roughly 2-fold increased in euchromatin for the error range of our experiment (0.75 < *c*_2_ < 1). For the average value *c*_2_ = 0.9 an 8-fold increased methylation rate of SUV39H in PCH is obtained.

### Mathematical modeling of the pericentric heterochromatin network

A chromatin fiber of 300 nucleosomes arranged on a linear 60 kb DNA fragment was modeled with a nucleosome repeat length of 200 bp. Each nucleosome on the fiber was able to contact the others via chromatin looping. The collision probability was determined as the local concentration of one nucleosome in the proximity of the others as described previously (Erdel et al, 2013; Rippe, 2001; Rippe et al, 1995). The resulting values for an increased local concentration of nucleosomes in the proximity of the first nucleosome at the 0-position due to chromatin looping is plotted in Fig 7A.

To represent the PCH state, we replaced every 8^th^ nucleosome of the uniform euchromatic fiber by a high affinity binding site, referred to as an ‘origin’ (parameter ‘*o*’ in the model). The total number of origins was determined by the model based on SUV39H concentration and the experimentally-derived association and dissociation rates of SUV39H (Supplementary Tables S2, S7) to yield 38 origins in a chain of 300 nucleosomes for the modeling work. The number of nucleation sites as represented by SUV39H-bound origins (‘*SHo*’) was determined from the amount of immobilized SUV39H compared to the nucleosome concentration in PCH. We computed that every 170^th^ nucleosome or every 21^st^ origin site was in a nucleation site state. For euchromatin the corresponding calculations yielded an origin site every 71 nucleosomes.

The mathematical model consists of a system of ordinary differential equations (ODEs) as shown below (equation 11). The core variables constituting the network (Fig 7B) are the local probability of methylation and the local probabilities of occupation by HP1, SUV39H and HP1-SUV39H complex that depend on time and the position of nucleosomes and nucleation origins on the fiber. The measured parameters for the three different HP1α/β/γ isoforms as well as for the SUV39H1 and SUV39H2 were integrated into a single ‘virtual’ HP1 or SUV39H protein, respectively. The model variables and parameters as well as reference values are given in Supplementary Table S7. The index *i* denotes the position of an individual nucleosome on the fiber. Nucleation origins positioned within the fiber were described by the first three equations. Their state transitions do not depend on the states of the adjacent nucleosomes and their position on the fiber.

$$\begin{matrix} \frac{do}{dt}=\text{k}_{-\text{h2}}H\text{o + k}_{-\text{s3}}S\text{o - }k_{+}\text{(}c_{\text{s}}+c_{\text{h}}\text{) o} \\ \frac{dSo}{dt}=k_{+}c_{\text{s}}\text{o + k}_{-\text{h4}}\text{(1 - o - So - Ho) - (}k_{+}c_{\text{h}}+\text{k}_{-\text{s3}}\text{)So } \\ \frac{dHo}{dt}=k_{+}c_{\text{h}}\text{o + k}_{-\text{s4}}\text{(1 - o - So - Ho) - (}k_{+}c_{\text{s}}+\text{k}_{-\text{h2}}\text{)Ho } \\ \frac{dn_{i}}{dt}=k_{-\text{m}}(1-n_{i}-Sm_{i}-Hm_{i}-SHm_{i})-n_{i}\left[k_{\text{u}}+k_{\text{m}}(c_{\text{s}}+\sum_{l=0}^{299} (SHm_{l}+Sm_{l})j_{\text{m}}(\left|i-l\right|)+(SHo+So)\sum_{k=0}^{37} j_{\text{m}}(\left|i-8k\right|))\right] \\ \frac{dHm_{i}}{dt}=k_{+}c_{\text{h}}(1-n_{i}-Sm_{i}-Hm_{i}-SHm_{i})+k_{-\text{s2}}SHm_{i}-(k_{+}c_{\text{s}}+k_{-\text{h2}})Hm_{i} \\ \frac{dSm_{i}}{dt}=k_{+}c_{\text{s}}(1-n_{i}-Sm_{i}-Hm_{i}-SHm_{i})+k_{-\text{h2}}SHm_{i}-(k_{+}c_{\text{h}}+k_{-\text{s1}})Sm_{i} \\ \frac{dSHm_{i}}{dt}=k_{+}c_{\text{s}}Hm_{i}+k_{+}c_{\text{h}}Sm_{i}-(k_{-\text{s2}}+k_{-\text{h2}})SHm_{i} \end{matrix}$$

(11)

For euchromatin, nucleation origins were neglected due to their low abundance. Thus, the equations for euchromatin read:

$\begin{matrix} \frac{dn_{i}}{dt}=k_{-\text{m}}(1-n_{i}-Sm_{i}-Hm_{i}-SHm_{i})-n_{i}\left[k_{\text{u}}+k_{\text{e}}+k_{\text{m}}(c_{\text{s}}+\sum_{l=0}^{299} (SHm_{l}+Sm_{l})j_{m}(\left|i-l\right|))\right] \\ \frac{dHm_{i}}{dt}=k_{+}c_{\text{h}}(1-n_{i}-Sm_{i}-Hm_{i}-SHm_{i})+k_{-\text{s2}}SHm_{i}-(k_{+}c_{\text{s}}+k_{-\text{h2}})Hm_{i} \\ \frac{dSm_{i}}{dt}=k_{+}c_{\text{s}}(1-n_{i}-Sm_{i}-Hm_{i}-SHm_{i})+k_{-\text{h2}}SHm_{i}-(k_{+}c_{\text{h}}+k_{-\text{s1}})Sm_{i} \\ \frac{dSHm_{i}}{dt}=k_{+}c_{\text{s}}Hm_{i}+k_{+}c_{\text{h}}Sm_{i}-(k_{-\text{s2}}+k_{-\text{h2}})SHm_{i}\text{ } \end{matrix}$ (12)

The steady state solutions for the PCH and euchromatin fiber systems as functions of HP1, JMJD2 and number of origins were numerically derived using Mathematica 9.0 (Wolfram Research). The Mathematica code of the model is provided as Supplementary Material. Following the chemical master equation formalism we converted the deterministic model into the corresponding stochastic model. Stochastic kinetic traces and steady state distributions were simulated with the Gillespie stochastic simulation algorithm (Gillespie, 2007) implemented in C++. Based on our experimental data set and quantities available from the literature data some model parameters were directly calculated and fixed (Supplementary Table S7). The remaining non-constrained parameters *k*_u_, *k*_m_ and *k*_e_ were determined from the experimentally measured PCH and euchromatin steady state H3K9me3 levels. The resulting parameter set quantitatively reproduced the H3K9me3 levels in wild-type PCH (38 %) and in *Suv39h* double knockout PCH (13 %) as well as in wild-type euchromatin (28 %) (Fig 3C). The modeled methylation degree included the summation over all nucleosomes within the chromatin fiber in the methylated state (*m, Hm, Sm, SHm*):

$M=\frac{100\sum_{l=0}^{299} (1-n_{l})}{300-Number (origins)}$. (13)

## Supplementary Tables

### Supplementary Table S1. FCS analysis of HP1α, HP1β, HP1γ and SUV39H1.

| **Protein**  Cell lines | **Compart-ment** | ***n*** | ***c*_GFP-protein_**  **(µM)** | ***c*_endog.protein_**  **(µM)** | ***D*_1_**  **(µm² s^-1^)** | ***α*_1_** | ***D*_2_**  **(µm^2^ s^-1^)** | ***α*_2_** | ***Σc*_endogenous_**  **(µM)** |
| --- | --- | --- | --- | --- | --- | --- | --- | --- | --- |
| GFP-**HP1α ^a^**  NIH-3T3 | Cyto ^b^ | 11 | 0.24 ± 0.04 | 0.06 ± 0.04 | 42.1 ± 4.3 ^d^ | 0.83 ± 0.05 ^d^ | – | – | Cyto ^e^:  0.5 ± 0.2  Eu ^e^:  18.8 ± 11.9  PCH ^e^:  40.8 ± 25.2 |
|  | Eu ^c^ | 19 | 1.5 ± 0.3 | 0.5 ± 0.3 | 13.9 ± 1.4 ^d^ | 0.81 ± 0.04 ^d^ | 0.38 ± 0.07 ^d^ | > 1 ^d^ |  |
|  | PCH | 18 | 2.2 ± 0.5 | 1.0 ± 0.7 | 7.0 ± 1.6 ^d^ | 0.88 ± 0.12 ^d^ | 0.09 ± 0.04 ^d^ | > 1 ^d^ |  |
| GFP-**HP1β ^a^**  NIH-3T3 | Cyto ^b^ | 10 | 0.13 ± 0.06 | 0.14 ± 0.09 | 43.7 ± 10.1 ^d^ | 0.74 ± 0.05 ^d^ | – | – |  |
|  | Eu ^c^ | 19 | 1.45 ± 0.31 | 3.7 ± 2.9 | 5.8 ± 1.4 ^d^ | 0.79 ± 0.06 ^d^ | 0.13 ± 0.04 ^d^ | > 1 ^d^ |  |
|  | PCH | 12 | 2.0 ± 0.9 | 11.2 ± 8.7 | 6.7 ± 1.1 ^d^ | 0.83 ± 0.08 ^d^ | 0.07 ± 0.02 ^d^ | > 1 ^d^ |  |
| GFP-**HP1γ ^a^**  NIH-3T3 | Cyto ^b^ | 68 | 0.15 ± 0.03 | 0.27 + 0.13 | 51.7 ± 4.1 | 0.65 ± 0.02 | – | – |  |
|  | Eu ^c^ | 53 | 1.8 ± 0.2 | 14.7 ± 11.5 | 6.0 ± 1.1 | 0.63 ± 0.01 | 0.11 ± 0.03 | > 1 |  |
|  | PCH | 22 | 3.6 ± 0.7 | 28.6 ± 23.6 | 4.9 ± 2.2 | 0.66 ± 0.05 | 0.05 ± 0.02 | > 1 |  |
| **SUV39H1**-GFP  NIH-3T3 | Cyto ^b^ | 34 | 0.04 ± 0.01 | 0. 35 ± 0.01 | 25.5 ± 5.2 | 0.70 ± 0.02 | – | – | 0. 35 ± 0.01 |
|  | Eu ^c^ | 21 | 0.69 ± 0.27 | 0.42 ± 0.22 | 17.6 ± 7.6 | 0.71 ± 0.08 | 0.13 ± 0.05 | > 1 | 0.42 ± 0.22 |
|  | PCH | 6 | 1.45 ± 0.65 | 3.0 ± 1.7 | 18.2 ± 9.0 | 0.65 ± 0.20 | 0.02 ± 0.01 | > 1 | 3.0 ± 1.7 |

FCS autocorrelation functions (ACF) of GFP-labeled HP1 or SUV39H1 were analyzed with an anomalous diffusion model to derive averaged parameters from the indicated number of experiments *n*. This yielded diffusion coefficients *D*, anomaly parameters *α* and the concentration of GFP-tagged protein *c*_GFP-Protein_. Endogenous SUV39H1 concentrations were determined from the ratio of SUV39H1-GFP and endogenous SUV39H1 measured by quantitative SDS-PAGE with Coomassie-staining, yielding a 0.75 ± 0.03-fold expression of SUV39H1-GFP as compared to endogenous SUV39H1. The endogenous HP1 concentrations were calculated from the ratio GFP-HP1 to endogenous HP1 measured on quantitative western blots. A 4.2 ± 0.3-fold overexpression of GFP-HP1α, a 0.92 ± 0.4-fold expression for GFP-HP1β and a 0.55 ± 0.22-fold expression of GFP-HP1γ as compared to endogenous HP1α/β/γ was determined. From the value of *c*_SUV39H1-GFP_ or *c*_GFP-HP1_ measured in the cytoplasm absolute concentrations in this cellular compartment were obtained. The concentration ratios of PCH and euchromatin were derived from the fluorescence intensities of the respective proteins measured in the cytoplasm, euchromatin and PCH by quantitative APD imaging. Errors correspond to 95 % confidence intervals.

^a^ NIH-3T3 GFP-HP1α/β/γ cell lines stably expressing GFP-tagged HP1 isoforms.

^b^ The data for HP1/SUV39H1 in the cytoplasm were fit with a one-component anomalous diffusion model.

^c^ A two-component anomalous diffusion model was required to fit the ACF of measurements within euchromatin and PCH. The faster-moving fraction with diffusion constant *D*_1_ displayed subdiffusion behavior (*α* < 1), as expected for transient binding and/or diffusion in the presence of obstacles. For the second fraction, intensity fluctuations originating from chromatin-bound molecules moving together with chromatin were very slow and displayed anomaly parameters *α* > 1 (Müller et al, 2009).

^d^ FCS measurements of HP1α and HP1β were published previously in (Müller et al, 2009). In the latter study, we used Alexa 488 maleimide with a too low literature value of *D* = 210 μm^2^·s^−1^ as a reference. From calibration measurements with well-defined Tetraspeck beads (Muller et al, 2008), we conclude that our previous FCS analysis of HP1α/β underestimated the diffusion coefficients by a factor of roughly 1.8. Additional measurements were conducted for concentration determination.

^e^ The amount of all HP1 isoforms within cytoplasm, euchromatin or heterochromatin was summed up to yield the concentration of the total pool of HP1 in the three different cellular compartments.

### Supplementary Table S2. FRAP analysis of GFP-tagged proteins.

| **Protein**  Cell line | **Chromatin** | ***n*** | ***D*_eff_**  **(µm² s^-1^)** | ***k*_off_ (s^-1^)** | ***k^*^*_on_ (s^-1^)** | ***K*_aff_** | ***Free* (%)** | ***Bound* (%)** | ***f*_im_ (%)** |
| --- | --- | --- | --- | --- | --- | --- | --- | --- | --- |
| GFP-**HP1α ^a, b^**  NIH-3T3 | Eu | 74 | 0.18 ± 0.02 | – | – | – | 98 ± 1 | – | 2 ± 1 |
|  | PCH | 59 | 0.9 ± 0.3 | 0.26 ± 0.06 | 0.7 ± 0.2 | 3.1 ± 1.2 | 24 ± 2 | 70 ± 2 | 6 ± 2 |
| GFP-**HP1β ^a, b^**  NIH-3T3 | Eu | 10 | 0.15 ± 0.04 | – | – | – | 97 ± 2 | – | 3 ± 2 |
|  | PCH | 8 | 0.4 ± 0.3 | 0.27 ± 0.17 | 1.7 ± 2.3 | 4.5 ± 2.4 | 19 ± 7 | 73 ± 7 | 8 ± 6 |
| GFP-**HP1β ^c^**  iMEF *Suv39h* dn | Eu/PCH | 21 | 0.4 ± 0.1 | – | – | – | 99 ± 1 | – | 3 ± 3 |
| **HP1β**-GFP **^a^**  iMEF wt | Eu | 28 | 0.03 ± 0.003 | – | – | – | 94 ± 1 | – | 6 ± 1 |
|  | PCH | 25 | 0.09 ± 0.01 | 0.044 ± 0.003 | 0.35 ± 0.05 | 6.9 ± 0.7 | 13 ± 1 | 76 ± 2 | 11 ± 1 |
| **HP1β**-GFP **^d^**  iMEF *Suv4-20h* dn | Eu | 10 | 0.05 ± 0.01 | – | – | – | 93 ± 1 | – | 7 ± 1 |
|  | PCH | 24 | 0.14 ± 0.02 | 0.08 ± 0.01 | 0.52 ± 0.11 | 6.2 ± 0.9 | 15 ± 1 | 79 ± 2 | 6 ± 1 |
| GFP-**HP1γ  ^a, b^**  NIH-3T3 | Eu | 13 | 0.19 ± 0.04 | – | – | – | 98 ± 2 | – | 2 ± 2 |
|  | PCH | 13 | 0.5 ± 0.2 | 0.5 ± 0.2 | 2.8 ± 2.1 | 4.9 ± 2.3 | 20 ± 7 | 74 ± 7 | 6 ± 4 |
| **JMJD2C**-GFP **^e^**  NIH-3T3 | Eu | 9 | 0.6 ± 0.3 | – | – | – | 98 ± 2 | – | 2 ± 2 |
|  | PCH | 7 | 0.4 ± 0.1 | – | – | – | 97 ± 3 | – | 3 ± 3 |
| **MBD1**-GFP **^a^**  NIH-3T3 | Eu | 10 | 0.05 ± 0.01 | – | – | – | 93 ± 4 | – | 7 ± 4 |
|  | PCH | 13 | 0.07 ± 0.04 | 0.02 ± 0.01 | 0.07 ± 0.03 | 2.8 ± 0.5 | 24 ± 3 | 66 ± 5 | 10 ± 5 |
| GFP-**MECP2 ^a^**  NIH-3T3 | Eu | 14 | 0.05 ± 0.01 | – | – | – | 98 ± 2 | – | 2 ± 2 |
|  | PCH | 14 | 0.2 ± 0.1 | 0.04 ± 0.02 | 0.2 ± 0.1 | 4.0 ± 0.7 | 17 ± 3 | 65 ± 8 | 18 ± 9 |
| **PAX3**-GFP **^e^**  NIH-3T3 | Eu | 15 | 0.5 ± 0.2 | – | – | – | 98 ± 4 | – | 2 ± 4 |
|  | PCH | 19 | 0.7 ± 0.3 | – | – | – | 98 ± 3 | – | 2 ± 3 |
| **SUV39H1**-GFP  NIH-3T3 | Eu | 53 | 0.5 ± 0.3 | 0.19 ± 0.1 | 0.5 ± 0.4 | 2.1 ± 0.3 | 32 ± 3 | 65 ± 3 | 3 ± 1 |
|  | PCH | 52 | 0.3 ± 0.1 | 0.06 ± 0.02 | 0.3 ± 0.1 | 4.0 ± 0.8 | 18 ± 3 | 62 ± 3 | 20 ± 5 |
| **SUV39H1**-GFP ^f^  iMEF *Suv39h* dn | Eu | 10 | 0.4 ± 0.2 | 0.2 ± 0.1 | 0.5 ± 0.3 | 2.6 ± 0.4 | 29 ± 4 | 71 ± 3 | 0 ± 1 |
|  | PCH | 7 | 0.2 ± 0.1 | 0.13 ± 0.03 | 0.32 ± 0.05 | 2.6 ± 0.3 | 27 ± 2 | 70 ± 1 | 4 ± 3 |
| **SUV39H1-H320R**-GFP ^g^  NIH-3T3 | Eu | 6 | 1.8 ± 1.4 | 0.2 ± 0.2 | 0.41 ± 0.28 | 2.3 ± 0.7 | 30 ± 6 | 66 ± 6 | 4 ± 5 |
|  | PCH | 8 | 0.2 ± 0.1 | 0.03 ± 0.01 | 0.14 ± 0.09 | 3.4 ± 1.6 | 20 ± 6 | 55 ± 6 | 25 ± 8 |
| **SUV39H1-H324L**-GFP ^g^  NIH-3T3 | Eu | 12 | 0.5 ± 0.5 | 0.14 ± 0.17 | 0.16 ± 0.11 | 2.5 ± 0.8 | 29 ± 6 | 68 ± 6 | 3 ± 3 |
|  | PCH | 8 | 0.04 ± 0.02 | 0.03 ± 0.02 | 0.13 ± 0.16 | 4.8 ± 5.4 | 27 ± 8 | 67 ± 8 | 6 ± 3 |
| **SUV39H2**-GFP  NIH-3T3 | Eu | 8 | 0.013 ± 0.008 | 0.004 ± 0.002 | 0.01 ± 0.01 | 2.3 ± 0.8 | 23 ± 6 | 52 ± 6 | 24 ± 13 |
|  | PCH | 28 | 0.014 ± 0.007 | 0.007 ± 0.002 | 0.04 ± 0.01 | 5.2 ± 1.3 | 9 ± 1 | 39 ± 1 | 52 ± 13 |
| **SUV4-20H1**-GFP **^a^**  iMEF | Eu | 10 | 0.11 ± 0.05 | – | – | – | 85 ± 2 | – | 15 ± 8 |
|  | PCH | 29 | 0.08 ± 0.04 | 0.04 ± 0.01 | 0.20 ± 0.11 | 4.1 ± 1.0 | 15 ± 2 | 54 ± 2 | 30 ± 5 |
| **SUV4-20H2**-GFP **^a^**  iMEF | Eu | 8 | 0.13 ± 0.10 | – | – | – | 92 ± 1 | – | 17 ± 11 |
|  | PCH | 23 | 0.011 ± 0.005 | 0.010 ± 0.004 | 0.05 ± 0.04 | 4.4 ± 1.1 | 8 ± 1 | 33 ± 1 | 59 ± 12 |

Fluorescence recovery after photobleaching (FRAP) measurements were fit with a diffusion, a reaction-diffusion and a reaction model. Parameters are given for the model that yielded the best fit. All measurements were performed in G1 and S phase cells and averaged over *n* experiments. Errors correspond to 95 % confidence intervals.

^a^ The mobility of MBD1, MECP2, SUV4-20H1/2-GFP and all isoforms of HP1-GFP in euchromatin of wild-type fibroblasts was very well described by a diffusion model. The effective diffusion coefficient *D*_eff_ includes low affinity/transient binding contributions. For PCH a reaction-diffusion model was required to describe the data for these proteins that additionally yielded the binding rate constants listed in the table. All other measurements were fit with a reaction-diffusion model.

^b^ NIH-3T3 GFP-HP1α/β/γ cell lines stably expressing GFP-tagged HP1 isoforms.

^c^ In the iMEF *Suv39h* dn cells HP1 did not colocalize with the chromocenters, although they were still present as evident from the DAPI staining or the H2A-RFP distribution. The results in euchromatin and PCH were well described by a pure diffusion model and were indistinguishable with respect to the values determined for *D*_eff_ and the immobile fraction. Accordingly, only the average value of measurements in different chromatin compartments is given for the iMEF *Suv39h* dn cells.

^d^ In *Suv4-20h* knockout cells HP1 still localized to PCH.

^e^ The mobility of JMJD2C-GFP and PAX3-GFP in euchromatin and PCH was well described by an effective diffusion model. Cells were co-transfected with H2A-RFP to identify PCH.

^f^ SUV39H1-GFP transfected into iMEF *Suv39h* dn cells was enriched at PCH in about 25% of the cells after 24 h. Chromocenters were identified by an H2A-RFP costaining. FRAP data are average values of randomly picked cells.

^g^ SUV39H1 mutants that were either hyperactive (H320R) or inactive (H324L) with respect to wild-type (Rea et al, 2000). These mutants showed no changes in protein-binding rates for class II and III binding. However, the immobilized protein fraction of SUV39H1 (wild-type: 20 ± 5%) was slightly larger for the hyperactive (25 ± 8%) and smaller for the inactive protein (6 ± 3%).

### Supplementary Table S3. Measured and calculated molecular weights and hydrodynamic parameters.

| **Complex** | ***M*_exp_^a^ (kDa)** | ***M*_calc_^b^ (kDa)** | ***S*_exp_^c^ (S)** | ***S*_calc_^d^ (S)** | ***D*_exp_^e^ (µm^2^ s^-1^)** | ***D*_calc_^d^ (µm^2^ s^-1^)** | **^b^ (ml g^-1^)** |
| --- | --- | --- | --- | --- | --- | --- | --- |
| **HP1β** | n. d. | 22.7 | 2.0 ± 0.1 | 1.9 | n. d. | 76 | 0.728 |
| **HP1β-HP1β** | 46.7 ± 3.2 | 45.4 | 2.8 ± 0.1 | 2.7 | 76 ± 8 | 52 | 0.728 |
| **CD** | 9.0 ± 0.2 | 9.4 | 1.4 ± 0.1 | 1.2 | n. d. | 104 | 0.731 |
| **CSD-CSD** | 21.0 ± 0.3 | 21.5 | 1.9 ± 0.2 | 2.2 | n. d. | 92 | 0.726 |

Molecular weights and hydrodynamic parameters of recombinant mouse His-tagged full-length HP1β and its isolated chromodomain (CD) and chromoshadow domain (CSD) in the concentration range from 8-30 µM. At these concentrations HP1β  formed a stable dimer via its CSD domain as apparent from the measured hydrodynamic parameters. Values refer to H2O and a temperature of 20 ºC as standard state. At low µM concentrations a *bona fide* monomeric HP1β species appeared at 2.0 ± 0.1 S with an equilibrium dissociation constant of 1-2 µM as determined from the relative molar fractions of monomer and dimer species (Fig 2A).

^a^ Determined by sedimentation equilibrium ultracentrifugation and data analysis with Ultrascan (Demeler, 2005). Equilibrium data sets from three different loading concentrations at three speeds were used for a global curve fitting analysis to a one-component model.

^b^ Calculated from amino acid composition with Sednterp (Laue et al, 1992) and a value of 0.546 ml g^-1^ for the partial specific volume of DNA.

^c^ Determined from the averaged peak in the sedimentation coefficient *c*(*s*)-distribution after sedimentation velocity ultracentrifugation.

^d^ Calculated from the pdb coordinates with Hydropro (Garcia De La Torre et al, 2000).

### Supplementary Table S4. Summary of protein-protein associations.

|  | **HP1α** | **HP1β** | **HP1γ** | **SUV39H1** | **SUV39H2** | **SUV4-20H1** | **SUV4-20H2** | **DNMT1** | **MBD1** | **MECP2** |
| --- | --- | --- | --- | --- | --- | --- | --- | --- | --- | --- |
| **HP1α** | + |  |  |  |  |  |  |  |  |  |
| **HP1β** | + | + |  |  |  |  |  |  |  |  |
| **HP1γ** | + | + | + |  |  |  |  |  |  |  |
| **SUV39H1** | + | + | + | + |  |  |  |  |  |  |
| **SUV39H2** | n.d. | n.d. | n.d. | n.d. | n.d. |  |  |  |  |  |
| **SUV4-20H1** | n.d. | n.d. | n.d. | n.d. | n.d. | n.d. |  |  |  |  |
| **SUV4-20H2** | + | + | + | n.d. | n.d. | – | + |  |  |  |
| **DNMT1^a^** | + | + | n.d. | + | n.d. | n.d. | n.d. | + |  |  |
| **MBD1^b,d^** | + | n.d. | n.d. | + | n.d. | n.d. | n.d. | n.d. | n.d. |  |
| **MECP2^c,d^** | + | + | + | + | n.d. | n.d. | n.d. | + | n.d. | n.d. |

Summary of protein-protein associations within the PCH network from FCCS and F2H experiments conducted in this study and supplemented with previously published data for MBD-containing proteins. The FCCS and F2H studies conducted here provide a direct readout for interaction in living mouse cells. For HP1β and its CD and CSD domains we also determined by analytical ultracentrifugation experiments that the protein forms a dimer via its CSD with a *K*_d_ of 1-2 µM for the dimerization and no higher order complex up to a concentration of 30 µM. Furthermore, the FCCS experiments demonstrated that all HP1 isoforms show homo- and heterodimerization. The HP1 interaction with either SUV39H1 or SUV4-20H2 was tested via the F2H assay and the F2H interaction analysis for SUV4-20H2 was conducted previously (Hahn et al, 2013). Interaction of HP1 and SUV39H1 with DNMT1, MBD1 and MECP2 were reported previously in the studies referenced below.

^a^ HP1: (Fuks et al, 2003; Lehnertz et al, 2003; Smallwood et al, 2007). SUV39H1: (Esteve et al, 2006; Fuks et al, 2003). DNMT1: (Fellinger et al, 2009)

^b^ HP1: (Fujita et al, 2003). SUV39H1: (Fujita et al, 2003)

^c^ HP1: (Agarwal et al, 2007). SUV39H1: (Lunyak et al, 2002). DNMT1: (Kimura & Shiota, 2003)

^d^ Tethering MBD1-GFP or GFP-MECP2 to the *lac*O-arrays resulted in the recruitment of RFP-HP1α but not of RFP-SUV39H1. Although interactions of MBD1/MECP2 and SUV39H1 have been shown in pulldown experiments (Fujita et al, 2003; Lunyak et al, 2002), this interaction was not reproduced in the F2H assay when testing for all possible combinations of N- or C-terminally tagged proteins.

### Supplementary Table S5. Perturbations of PCH features.

|  | **DAPI-**  **dense DNA** | **5meC** | **DNMT1** | **MECP2** | **MBD1** | **H3K9me3** | **SUV39H1/2** | **HP1α/β/γ** | **H4K20me3** | **SUV4-20H1/2** |
| --- | --- | --- | --- | --- | --- | --- | --- | --- | --- | --- |
| **Wild-type** | + | + | + | + | + | + | + | + | + | + |
| ***Suv39h1/2***  **double null^a^** | + | + | +^b^ | +^b^ | +^b^ | – | dn^a^ | – | –^c^ | –^c^ |
| **JMJD2B overexpr.** | + | n.d. | n.d. | n.d. | n.d. | –^d^ | n.d. | n.d. | n.d. | n.d. |
| ***Suv4-20h1/2***  **double null** | + | n.d. | n.d. | n.d. | n.d. | + | + | + | –^e^ | dn^e^ |
| **TSA histone acetylation** | – ^f^ | n.d. | n.d. | n.d. | n.d. | – ^f^ | – | – ^f^ | n.d. | n.d. |
| **HP1α/β/γ**  **knock down** | + | n.d. | n.d. | n.d. | n.d. | – | – | k.d. | n.d. | n.d. |
| **G2 phase** | + | n.d. | + | n.d. | n.d. | + | + | – | + | + |

The following perturbations were considered: (i) Varying the histone methylation levels of H3K9 and H4K20 by knockout of the responsible methylases or alternatively by JMJD2 overexpression, (ii) inhibiting histone methylation by enhanced acetylation due to inhibition of deacetylation via TSA, (iii) manipulating the abundance of potential interaction partners like HP1 in knockdown experiments, (iv) loss of HP1 due to H3 serine 10 phosphorylation and H3 lysine 14 acetylation during G2 phase. These parameters affected protein binding and localization to reveal interdependencies of the network. The ‘**+’** designation shows that the PCH condensation state, DNA or histone modifications or protein binding remained similar to the wild-type state. In contrast, ‘**–‘** indicates significant alterations. References to observations made already in previous publications are given below.

^a^ The effects of both SUV39H1 and SUV39H2 deficiency were initially described in (Peters et al, 2001)

^b^ DNMT1, MBD1 and MECP2 remained localizing at PCH as shown in (Brero et al, 2005; Lehnertz et al, 2003) and in Supplementary Fig S2B

^c^ Loss of SUV4-20H and H4K20me3 from PCH was shown previously (Schotta et al, 2004)

^d^ Due to overexpression of the H3K9me3-specific demethylase JMJD2C the H3K9me3 level was drastically reduced (Fodor et al, 2006). This effect was quantitated here (Fig 8C).

^e^ *Suv4-20h* dn cells and their effect on H4K20me3 were published by Schotta et al. (Schotta et al, 2008)

^f^ According to Fig S2D and previous references (Fejes Tóth et al, 2004; Maison et al, 2002; Taddei et al, 2001) histone hyperacetylation induced decondensation of heterochromatin/PCH.

### Supplementary Table S6. Cell cycle-dependent mobility of SUV39H1 and HP1α.

| **Protein**  Cell line | **Cell cycle** | **Chromatin** | ***n*** | ***D*_eff_ (µm² s^-1^)** | ***k*_off_ (s^-1^)** | ***k^*^*_on_ (s^-1^)** | ***K*_aff_** | ***Free* (%)** | ***Bound* (%)** | ***f*_im_ (%)** |
| --- | --- | --- | --- | --- | --- | --- | --- | --- | --- | --- |
| GFP-**HP1α **  NIH-3T3 | G1 | Eu^a^ | 10 | 0.24 ± 0.06 | – | – | – | 98 ± 2 | – | 2 ± 2 |
|  |  | PCH ^b^ | 9 | 0.9 ± 0.6 | 0.22 ± 0.09 | 0.6 ± 0.2 | 2.7 ± 0.8 | 27 ± 6 | 67 ± 6 | 6 ± 3 |
|  | S | Eu ^a^ | 9 | 0.18 ± 0.03 | – | – | – | 95 ± 3 | – | 5 ± 3 |
|  |  | PCH ^b^ | 12 | 1.1 ± 0.5 | 0.22 ± 0.04 | 0.7 ± 0.1 | 3.1 ± 0.5 | 24 ± 3 | 71 ± 3 | 5 ± 4 |
|  | G2 | Eu ^a^ | 14 | 0.17 ± 0.04 | – | – | – | 99 ± 1 | – | 1 ± 1 |
|  |  | PCH ^b^ | 18 | 0.09 ± 0.02 | – | – | – | 97 ± 2 | – | 3 ± 2 |
| **SUV39H1**-GFP  NIH-3T3 | G1 | Eu | 5 | 0.3 ± 0.4 | 0.12 ± 0.10 | 0.2 ± 0.2 | 1.5 ± 0.03 | 40 ± 0 | 59 ± 0 | 1 ± 1 |
|  |  | PCH | 5 | 0.5 ± 0.1 | 0.08 ± 0.03 | 0.3 ± 0.2 | 3.4 ± 1.0 | 22 ± 5 | 71 ± 5 | 7 ± 7 |
|  | S | Eu | 11 | 0.7 ± 0.7 | 0.6 ± 0.6 | 1.7 ± 2.4 | 2.5 ± 0.7 | 28 ± 5 | 69 ± 5 | 3 ± 2 |
|  |  | PCH | 11 | 0.5 ± 0.4 | 0.15 ± 0.06 | 0.6 ± 0.2 | 3.7 ± 0.5 | 18 ± 3 | 65 ± 3 | 17 ± 8 |
|  | G2 | Eu | 13 | 1.1 ± 0.7 | 0.2 ± 0.1 | 0.9 ± 1.1 | 3.0 ± 1.5 | 29 ± 6 | 68 ± 6 | 3 ± 3 |
|  |  | PCH | 12 | 0.3 ± 0.2 | 0.05 ± 0.01 | 0.3 ± 0.3 | 5.3 ± 2.8 | 20 ± 7 | 69 ± 7 | 11 ± 5 |
|  | M ^c^ | mitotic chromatin | 11 | 6 ± 5 | 1.2 ± 0.4 | 3 ± 1 | 2.4 ± 0.2 | 28 ± 2 | 67 ± 2 | 5 ± 3 |

FRAP measurements were assigned to cell cycle phases via PCNA-based identification of G1 and S phase. Cells in G2 phase were identified via delocalization of co-transfected RFP-HP1α. Values refer to the average of *n* experiments. For SUV39H1 no significant cell cycle-dependent diff­erences in mobility were detected. For HP1α an increased mobility in PCH was observed in G2. Errors correspond to 95 % confidence intervals.

^a^ In euchromatin the GFP-HP1 mobility is well described by a diffusion-dominant model. Here, the effective diffusion coefficient *D*_eff_ also includes low affinity/transient binding contributions of HP1.

^b^ For PCH a reaction-diffusion model was applied to yield the diffusion coefficient as well as the kinetic rate constants for binding.

^c^ Due to the high mobility of mitotic cells, average values were associated with relatively high error ranges.

### Supplementary Table S7. Parameters used for modeling the PCH network.

| **Parameter** | **Value** | **Comment** |
| --- | --- | --- |
| Nuclear volume | 3259 ± 85 µm^3^ | NIH-3T3 mouse fibroblast cells (Cantaloube et al, 2012) |
| Mouse genome size | 5·10^9^ bp | G1, diploid |
| PCH (fraction of major satellite rep.) | 3.6 % | Mouse genome sequencing, percentage of reads (Waterston et al, 2002) |
| Number of chromocenters | 28 ± 1 | DAPI/HP1 staining and automated 3D microscopy analysis (Cantaloube et al, 2012) |
| Average genome content of single chromocenter | 6.4 Mb | PCH genome fraction and number of chromocenters |
| Total volume of all chromocenters | 75 ± 2 µm^3^ | DAPI/HP1 staining and automated 3D microscopy analysis (Cantaloube et al, 2012) |
| Average volume of single chromocenter | 2.69 ± 0.04 µm^3^ | Chromocenter volume and number of chromocenters |
| PCH volume fraction | 2.31 ± 0.06 % | Nuclear volume fraction of chromocenter volume |
| Eu volume fraction | 97.69 ± 0.06 % | 100 % minus PCH volume fraction |
| PCH compaction ratio | 1.8 ± 0.3-fold | H2A-RFP fluorescence PCH vs. Eu (Fig 1B) |
| Nucleosome repeat length (NRL) | 191.1 ± 0.5 bp | MNase-seq in mouse fibroblasts (Teif et al, 2012) |
| Nucleosomes per nucleus | (26.2 ± 0.1)·10^6^ | Genome size / NRL |
| Nucleosome conc. average | 134 ± 4 µM | Number of nucleosomes / nuclear volume |
| Nucleosome conc. PCH | 234 ± 4 µM | From PCH compaction and PCH genome fraction |
| Nucleosome conc. Eu | 130 ± 4 µM | Remaining 96.4 % genome fraction |

| **Parameter** | **Value** | | **Comment** |
| --- | --- | --- | --- |
| H3K9me3 average | 28 ± 2 % | | Mass spectrometry NIH-3T3 (Fodor et al, 2006) |
| H3K9me3 wt PCH | 38 ± 5 % | | Fig 3C |
| H3K9me3 wt Eu | 28 ± 2 % | | Fig 3C |
| H3K9me3 *Suv39h* dn PCH | | 13 ± 2 % | Fig 3C |
| H3K9me3 *Suv39h* dn Eu | 25 ± 4 % | | Fig 3C |
| Concentration free HP1 dimer | 1.5 µM | | *c*_h_, calculated from FCS and FRAP data as described above in “Measurements of endogenous protein concentrations…” |
| Concentration free SUV39H dimer | 0.05 µM | | *c*_s_, calculated from FCS and FRAP data as described above in “Measurements of endogenous protein concentrations…” |
| Total nuclear concen­tration HP1 dimer | 9.8 µM | | Average of measured PCH and Eu concentrations weighted according to nuclear volume fraction |
| Total nuclear concen­tration SUV39H dimer | 0.33 µM | | Average of measured PCH and Eu concentrations weighted according to nuclear volume fraction |
| Specificity ratio SUV39H | > 8.3 | | Specificity ratio of PCH vs. euchromatin for SUV39H according to Fig 3D |
| Specificity ratio G9A/SETDB1 | < 2.2 | | Specificity ratio of euchromatin vs. PCH for histone methylases G9A and SETDB1 according to Fig 3D |
| Methylation by SUV39H | 4.8·10^-4^ µM^-1^ min^-1^ | | *k*_m_, from fit to measured methylation levels |
| Unspecific methylation | 1.8·10^-4^ min^-1^ | | *k*_u_, from fit to measured H3K9me3 methylation that occurs both in PCH and euchromatin |
| Euchromatic methylation | 2.6·10^-4^ min^-1^ | | *k*_e_, from fit to measured H3K9me3 methylation that is specific to euchromatin by G9A/SETDB1 |
| Demethylation | 13·10^-4^ min^-1^ | | *k*_-m_, from mass spectrometry experiments in HeLa cells (Zee et al, 2010) |
| Chromatin association rate of SUV39H and HP1 | 1.05 µM^-1^ min^-1^ | | *k*_+_, estimated parameter based on class II binding, see above “Estimation of kinetic association rate...” |
| *k*_off_ HP1 class II | 20 min^-1^ | | *k*_-h2_, experimentally determined parameter |
| *k*_off_ HP1 class IV | 0.5 min^-1^ | | *k*_-h4_, experimentally determined parameter |
| *k*_off_ SUV39H class I | 120 min^-1^ | | *k*_-s1_, experimentally determined parameter |
| *k*_off_ SUV39H class II | 11.3 min^-1^ | | *k*_-s2_, experimentally determined parameter |
| *k*_off_ SUV39H class III | 2.0 min^-1^ | | *k*_-s3_, experimentally determined parameter |
| *k*_off_ SUV39H class IV | 0.25 min^-1^ | | *k*_-s4_, experimentally determined parameter |
| SUV39H looping conc. | 0-14 µM | | *j*_M_, from chromatin bound SUV39H (Fig 7A) |

| **Parameter** | | **Value** | **Comment** |
| --- | --- | --- | --- |
| Nucleosomes without H3K9me3 | 72 % | | Variable *n*, recovered from model |
| Nucleosomes with H3K9me3 | 28 % | | Variable *m,* recovered from model with H3K9me3 averages of 38% in PCH and 28% in euchromatin |
| HP1-bound *m* nucleosome | 5.7 µM | | Variable *Hm*, concentration calculated from model based on FRAP binding rates |
| SUV39H-bound *m* nucleosome | 0.03 µM | | Variable *Sm*, concentration calculated from model based on FRAP binding rates |
| HP1-SUV39H *m* complex | 0.01 µM | | Variable *SHm*, concentration calculated from model based on FRAP binding rates |
| SUV39H methylation origins in PCH (free plus occupied) | 29.6 µM | | Variable *o*, concentration calculated from model based on FRAP binding rates |
| HP1-bound origins in PCH | 2.0 µM | | Variable *Ho*, concentration calculated from model based on FRAP binding rates |
| SUV39H-bound origins in PCH | 0.6 µM | | Variable *So*, concentration calculated from model based on FRAP binding rates |
| HP1-SUV39H origin complex in PCH | 1.4 µM | | Variable *SHo*, concentration of stably bound SUV39H dimer (class IV) |

The values given for the parameters listed in the table and used for the modeling in Fig 7 and Fig 8 were either determined in this study or taken from the indicated references. Measured parameters for the concentration and chromatin interactions of the three different HP1 isoforms as well as for the SUV39H1 and SUV39H2 were integrated into a single ‘virtual’ HP1 or SUV39H protein, respectively. For protein concentrations the isoform values were added. For kinetic off rates, weighted averages according to relative abundance are given.

## Supplementary Figures


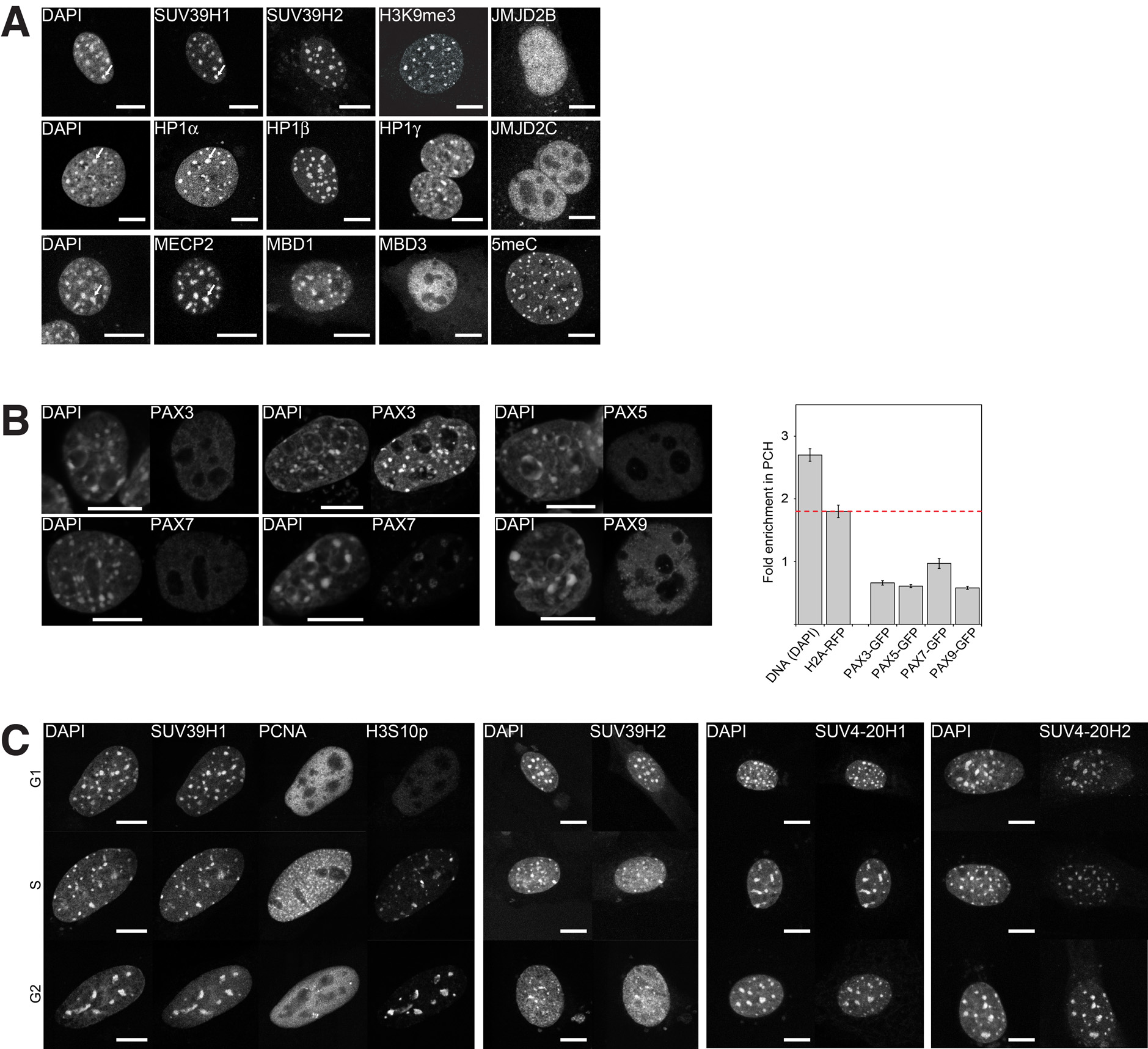


### Supplementary Figure S1. CLSM images of nuclear localization of PCH factors in NIH-3T3 mouse embryonic fibroblasts.

The colocalization of GFP-tagged proteins or histone/DNA modifications visualized by immunostaining with PCH foci identified from DAPI staining was evaluated. Scale bars, 10 µm.

1. The H3K9me3 mark and the responsible histone methylases SUV39H1 and SUV39H2 colocalized with the HP1 isoforms at PCH. In contrast, the H3K9-specific histone demethylases JMJD2B and JMJD2C were homogeneously distributed throughout the whole nucleus, showing no clear binding preference for euchromatin or PCH. Immunostaining for 5-methylcytosine (5meC) demonstrated the localization of highly methylated DNA in PCH regions. MECP2 and MBD1, both binding to methylated DNA via their MBD, followed the DNA methylation pattern in PCH. MBD3, which despite having an MBD-domain is unable to bind methylated DNA, displayed a mostly euchromatic localization as reported previously (Hendrich & Bird, 1998). In general, proteins that localized to PCH displayed this behavior for all foci present in a given cell. Accordingly, they also colocalize with the other factors enriched at these sites.
2. Nuclear distribution of transcription factors PAX3, PAX5, PAX7 and PAX9 (Bulut-Karslioglu et al, 2012) that have been proposed to recruit heterochromatin factors to PCH via DNA sequence-specific binding. For all GFP-tagged PAX3/5/7/9 factors a rather homogeneous distribution was observed. PAX3 and PAX7 enrichment at PCH was only observed for a subgroup of cells with high over-expression levels (about 5% and 29%, respectively). PAX5 was used as a negative control since PCH does not contain a PAX5 binding site. Error bars correspond to SD.
3. Both SUV39H and SUV4-20H histone lysine methylases remained associated with chromatin during G1, S and G2 cell-cycle phases. SUV4-20H1 and SUV4-20H2 as well as the H4K20me3 modification set by these enzymes were enriched in PCH.


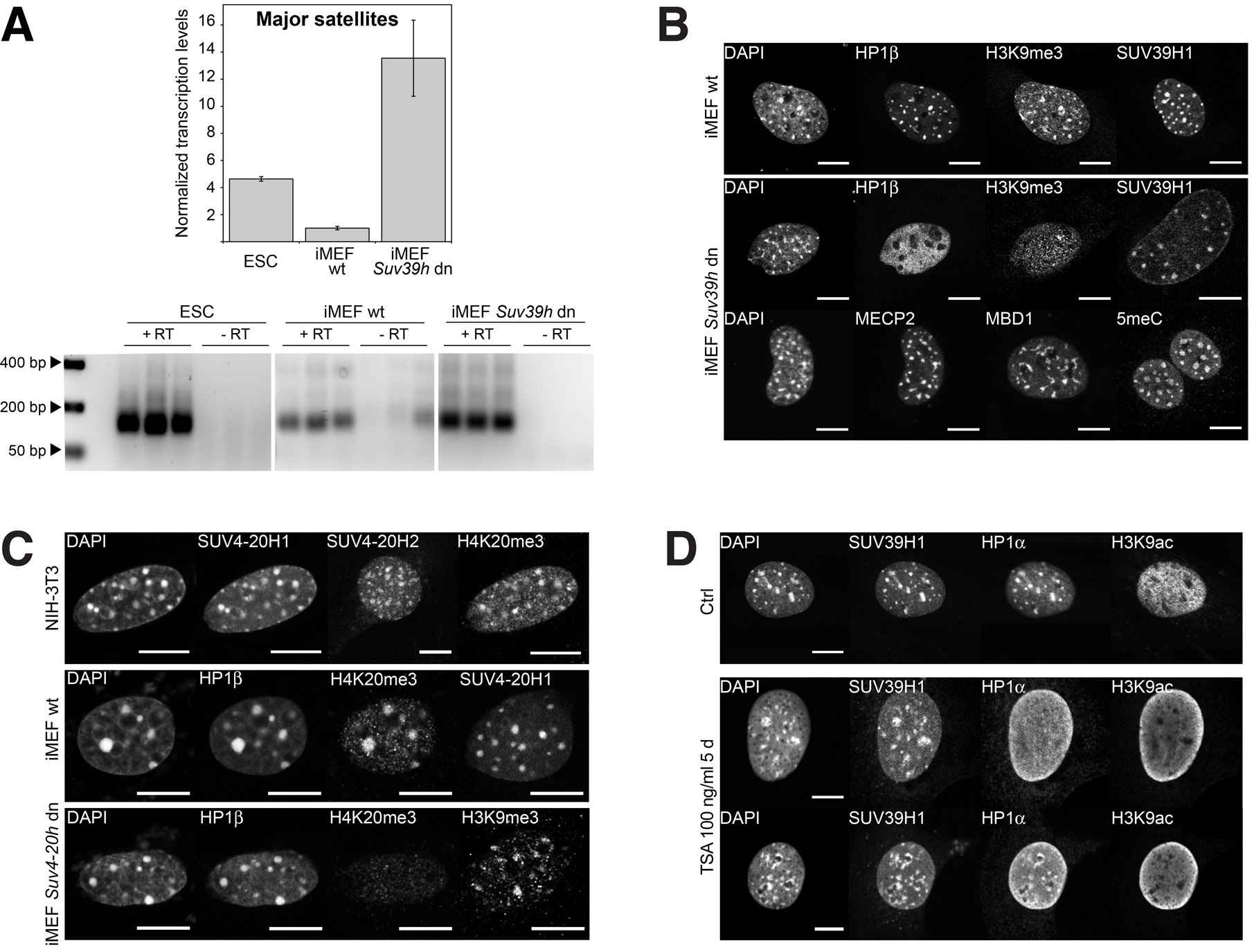


### Supplementary Figure S2. Major satellite repeat transcription measured by RT-qPCR and CLSM analysis of PCH organization in different cell types and knock-out cell lines.

1. Quantification of major satellite repeat transcription levels by RT-qPCR in ESCs, iMEF wt cells and iMEF *Suv39h* dn cells. Transcription levels were about 4.6 ± 0.2-fold higher for major satellites in ESCs as compared to fully differentiated iMEF wt cells. Furthermore, transcription levels further increased to 14 ± 2-fold for major repeats in iMEF *Suv39h* dn as compared to iMEF wt. The PCR products from the negative control samples (‘– RT’, produced without reverse transcriptase) were used to confirm the absence of DNA contaminations. Some residual DNA was present in the third ‘– RT’ iMEF sample, and thus this sample was not included in the quantification. Error bars correspond to SD.
2. Distribution of GFP-HP1β, H3K9me3 and SUV39H1-GFP relative to PCH in *Suv39h* dn cells that lost H3K9me3 and HP1β from PCH in agreement with previous reports (Müller et al, 2009; Peters et al, 2001). In contrast, the MBD-proteins MECP2 and MBD1 as well as 5meC remained enriched in PCH. As shown previously (Schotta et al, 2004), SUV4-20H and H4K20me3 were lost from the chromocenters in iMEF *Suv39h* dn cells. PCH chromatin compaction in the DAPI dense chromocenters persisted in the absence of *Suv39h* and the enrichment of H3K9me3.
3. Distribution of HP1β, SUV4-20H1/2 and H4K20me3 in knockout iMEFs of both SUV4-20H isoforms. In *Suv4-20h* dn cells PCH foci lost H4K20me3 but HP1 and H3K9me3 remained enriched in the chromocenters.
4. Changes of PCH organization in NIH-3T3 cells due to inhibition of histone deacetylation by trichostatin A (TSA) treatment for 5 days induced partial PCH decondensation, with some cells showing an almost complete HP1 loss (upper panel), while in others HP1 was still present at PCH in significant amounts (lower panel). For SUV39H1 only a moderate loss of the protein from PCH was observed in response to histone hyperacetylation.

**
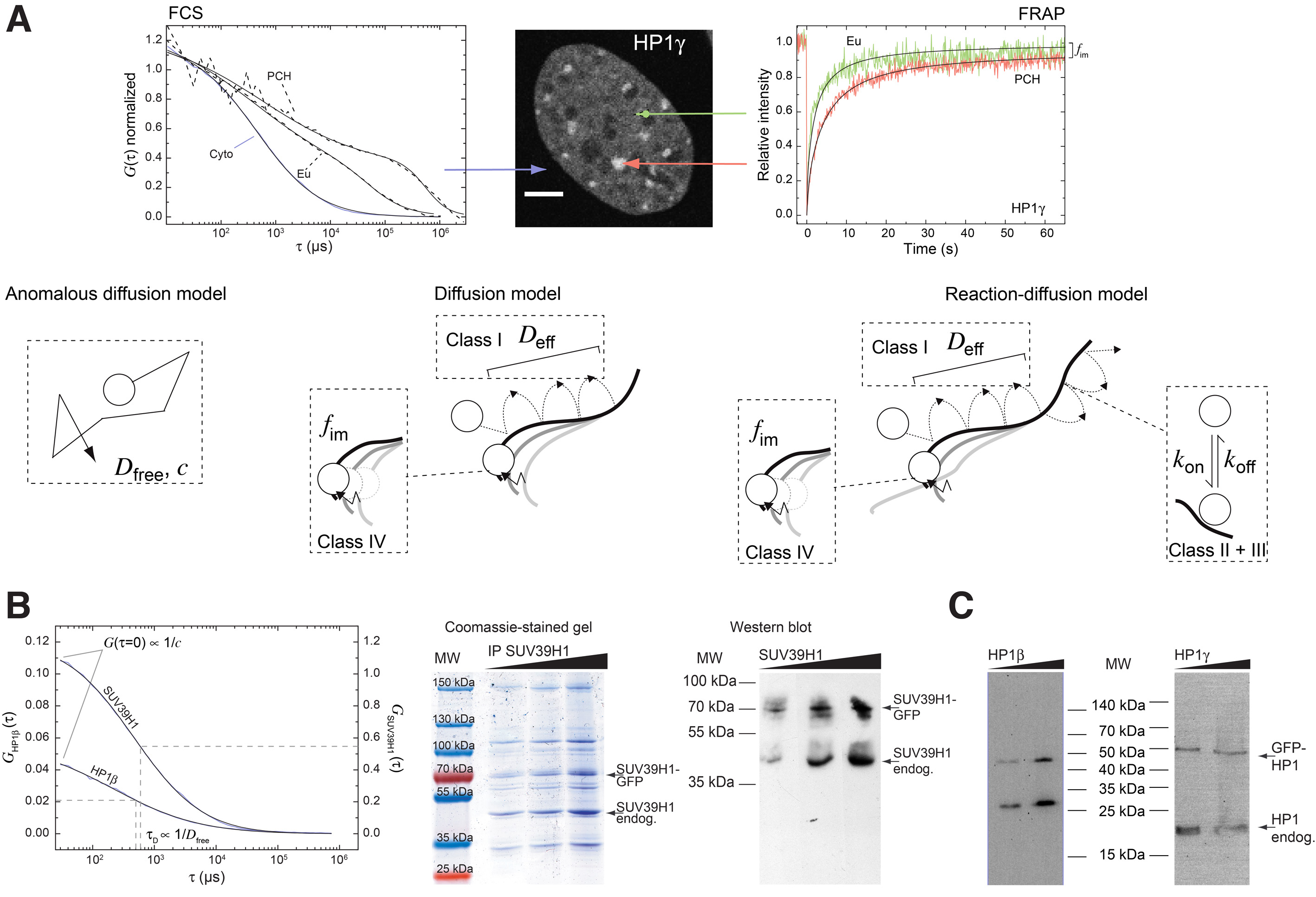
**

### Supplementary Figure S3. Quantification of protein parameters in living cells by FCS and FRAP

1. Approach to determine protein mobility and chromatin interaction parameters by combining FCS and FRAP experiments at different intracellular locations. In FCS the signal intensity fluctuations of diffusing molecules are recorded and evaluated by computing the autocorrelation function depicted on the top left side. It provides the concentration *c* of fluorescently labeled molecules and the diffusion coefficient *D* as a direct readout (see also panel B). FCS measurements in the cytoplasm provide a reference value for the diffusion coefficient *D*_free_ in the absence of chromatin binding. Reduced mobility due to binding interactions in euchromatin and PCH is reflected in a longer diffusion time *τ*. FRAP experiments record the fluorescence signal before and after a high laser intensity bleach to measure the time dependence of the fluorescence signal recovery. By fitting the integrated intensity data of the bleached region to a diffusion or a reaction-diffusion model the characteristic parameters for different binding classes can be extracted: the effective diffusion coefficient *D*_eff_ that includes transient binding interactions, the kinetic on- and off-rates (*k*_on_, *k*_off_) of chromatin binding events and the protein fraction *f*_im_ that is immobile during the experiment. The minimal number of binding site classes needed to rationalize the data in a consistent manner was extracted from the parameters measured by FCS and FRAP as indicated. Scale bar, 10 µm.
2. Determination of endogenous protein concentrations. The amplitude of the FCS autocorrelation function is inversely proportional to the concentration of fluorescently labeled molecules. Experimental curves for HP1β  and SUV39H1 in the cytoplasm are shown. FCS measurements in the cytoplasm were fit with a one-component anomalous diffusion model to extract the concentration of GFP-tagged protein in the cytoplasm. The endogenous protein concentrations were determined from the ratio of GFP-tagged protein to endogenous protein in a stably transfected cell line via Coomassie-stained SDS gels or quantitative western blots. Based on the quantification of immunoprecipitated as shown for SUV39H1(-GFP) the ratio of endogenous to labeled protein was determined and the endogenous protein concentration in the cytoplasm was calculated. The protein concentration ratios to PCH and euchromatin were derived from quantitative APD imaging, which determines average fluorescence intensities of the respective proteins in the different cellular compartments.
3. Determination of ratios of GFP-tagged to endogenous proteins for HP1β and HP1γ  by western blot analysis. Corresponding values for HP1α were determined previously (Müller et al, 2009).

**
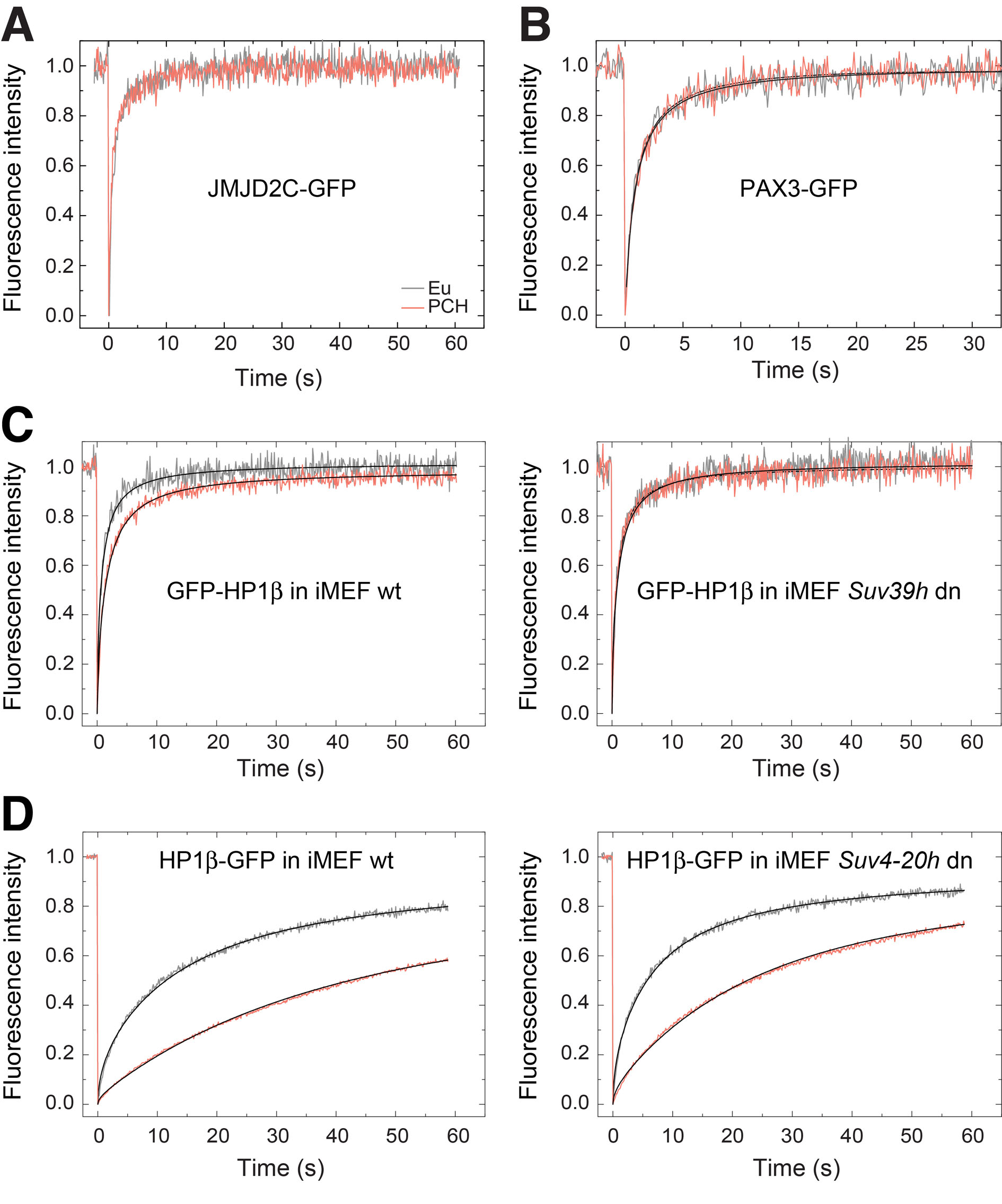
**

### Supplementary Figure S4. FRAP analysis of JMJD2C, PAX3 and HP1β in *Suv39h* dn and *Suv4-20h* dn cells.

1. FRAP measurements of JMJD2C-GFP mobility in PCH in comparison to euchromatin. An NIH-3T3 cell line stably expressing H2A-RFP to identify PCH foci was transfected with JMJD2C-GFP, which was distributed homogeneously in the nucleus. The JMJD2C mobility was similar in both compartments as evident from the overlay plot of the two curves.
2. Same as panel A but for PAX3-GFP, which also displayed a mostly homogeneous nuclear distribution. FRAP measurements in PCH versus euchromatin showed low chromatin binding affinity and a comparable protein mobility in both compartments.
3. FRAP measurements of HP1β in iMEF *Suv39h* dn cells. While HP1β−chromatin interactions in wt iMEFs (left panel) showed PCH-specific high-affinity binding, this feature was absent in *Suv39h* dn cells (right panel). For the latter, the recovery curves in PCH loci identified by H2A-RFP were well described by a diffusion model that yielded mobility values identical to those determined in euchromatic regions of wt iMEFs (Table S2).
4. FRAP measurements of HP1β-GFP in *Suv4-20h* dn iMEFs that lack both H4K20 methylases SUV4-20H1 and SUV4-20H2 in comparison to wt iMEFs. The mobility of HP1β in both euchromatin and PCH increased slightly while the immobile fraction was reduced in the *Suv4-20h* dn cells.

**
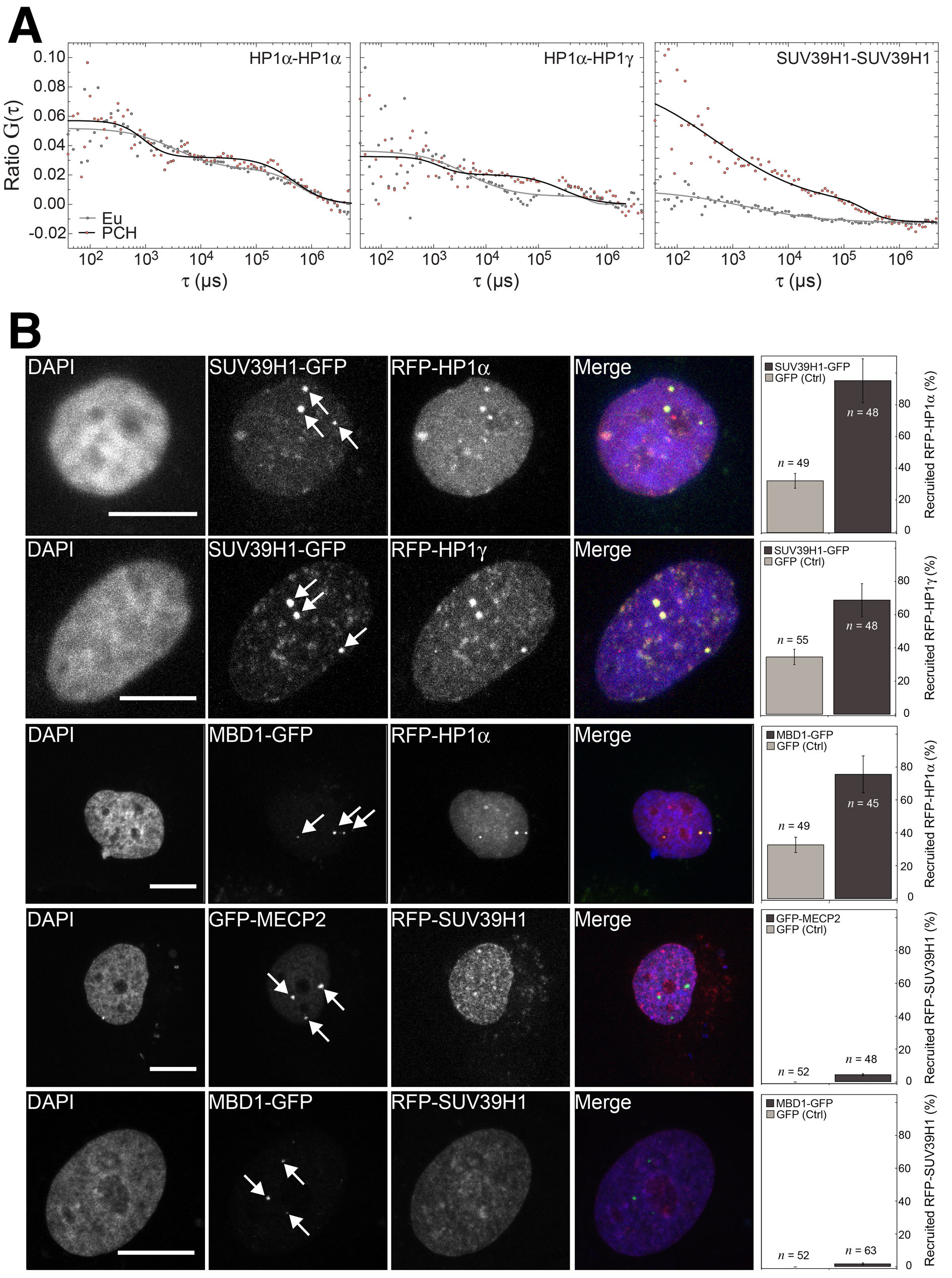
**

### Supplementary Figure S5. Protein-protein interaction analysis of HP1 and SUV39H1 by FCCS and F2H experiments.

1. FCCS measurements of interactions between HP1α and HP1γ and SUV39H1 (see Fig 2B for additional data). Quantitative analysis of FCCS curves revealed that most of the mobile HP1 pool existed as homo- or heterodimers. Furthermore, the FCCS experiments revealed self-association of soluble SUV39H1 in living cells. The soluble *bona fide* dimeric SUV39H1 protein fraction was calculated to comprise 24 ± 23 % of soluble SUV39H1 protein present at 0.34 µM concentration.
2. F2H analysis of SUV39H1 and HP1 protein-protein interactions (see Fig 2C for additional data). All HP1 isoforms interacted with SUV39H1 in living cells with the percentage of colocalizations indicated in the histogram in comparison to a negative control represented by the isolated GFP domain. The relatively high residual degree of colocalization of HP1**α** and HP1**γ** in the controls probably reflects the enrichment of these proteins at the *lac*O-array in the absence of ectopic tethering. Self-association of SUV39H1 was clearly demonstrated by the F2H assay. Furthermore, both MECP2 and MBD1 recruited HP1 efficiently, while the interaction of RFP-SUV39H1 was not observed with the MBD proteins, MECP2 and MBD1. Variation of N- and C-terminal fluorescent labels in all possible combinations did not influence this result. Scale bars, 10 µm. Error bars show the SD.

## Supplementary References

Agarwal N, Hardt T, Brero A, Nowak D, Rothbauer U, Becker A, Leonhardt H, Cardoso MC (2007) MeCP2 interacts with HP1 and modulates its heterochromatin association during myogenic differentiation. *Nucleic Acids Res* **35:** 5402-5408

Bacia K, Petrasek Z, Schwille P (2012) Correcting for Spectral Cross-Talk in Dual-Color Fluorescence Cross-Correlation Spectroscopy. *Chemphyschem* **13:** 1221-1231

Berg OG, von Hippel PH (1985) Diffusion-controlled macromolecular interactions. *Annu Rev Biophys Biophys Chem* **14:** 131-160

Brero A, Easwaran HP, Nowak D, Grunewald I, Cremer T, Leonhardt H, Cardoso MC (2005) Methyl CpG-binding proteins induce large-scale chromatin reorganization during terminal differentiation. *J Cell Biol* **169:** 733-743

Bulut-Karslioglu A, Perrera V, Scaranaro M, de la Rosa-Velazquez IA, van de Nobelen S, Shukeir N, Popow J, Gerle B, Opravil S, Pagani M, Meidhof S, Brabletz T, Manke T, Lachner M, Jenuwein T (2012) A transcription factor-based mechanism for mouse heterochromatin formation. *Nat Struct Mol Biol* **19:** 1023-1030

Cantaloube S, Romeo K, Le Baccon P, Almouzni G, Quivy JP (2012) Characterization of chromatin domains by 3D fluorescence microscopy: An automated methodology for quantitative analysis and nuclei screening. *BioEssays* **34:** 509-517

Chung I, Leonhardt H, Rippe K (2011) De novo assembly of a PML nuclear subcompartment occurs through multiple pathways and induces telomere elongation. *J Cell Sci* **124:** 3603-3618

Demeler B (2005) UltraScan - a comprehensive data analysis software package for analytical ultracentrifugation experiments. In *Modern Analytical Ultracentrifugation: Techniques and Methods*, Scott DJ, Harding SE, Rowe AJ (eds), pp 210-229. Cambridge: The Royal Society of Chemistry

Efroni S, Duttagupta R, Cheng J, Dehghani H, Hoeppner DJ, Dash C, Bazett-Jones DP, Le Grice S, McKay RD, Buetow KH, Gingeras TR, Misteli T, Meshorer E (2008) Global transcription in pluripotent embryonic stem cells. *Cell Stem Cell* **2:** 437-447

Erdel F, Müller-Ott K, Baum M, Wachsmuth M, Rippe K (2011) Dissecting chromatin interactions in living cells from protein mobility maps. *Chromosome Res* **19:** 99-115

Erdel F, Müller-Ott K, Rippe K (2013) Establishing epigenetic domains via chromatin-bound histone modifiers. *Ann N Y Acad Sci* **1305:** 29-43

Erdel F, Schubert T, Marth C, Langst G, Rippe K (2010) Human ISWI chromatin-remodeling complexes sample nucleosomes via transient binding reactions and become immobilized at active sites. *Proc Natl Acad Sci USA* **107:** 19873-19878

Esteve PO, Chin HG, Smallwood A, Feehery GR, Gangisetty O, Karpf AR, Carey MF, Pradhan S (2006) Direct interaction between DNMT1 and G9a coordinates DNA and histone methylation during replication. *Genes Dev* **20:** 3089-3103

Fejes Tóth K, Knoch TA, Wachsmuth M, Stöhr M, Frank-Stöhr M, Bacher CP, Müller G, Rippe K (2004) Trichostatin A induced histone acetylation causes decondensation of interphase chromatin. *J Cell Sci* **117:** 4277-4287

Fejes Tóth K, Mazurkiewicz J, Rippe K (2005) Association states of the nucleosome assembly protein 1 and its complexes with histones. *J Biol Chem* **280:** 15690-15699

Fellinger K, Rothbauer U, Felle M, Langst G, Leonhardt H (2009) Dimerization of DNA methyltransferase 1 is mediated by its regulatory domain. *J Cell Biochem* **106:** 521-528

Fodor BD, Kubicek S, Yonezawa M, O'Sullivan RJ, Sengupta R, Perez-Burgos L, Opravil S, Mechtler K, Schotta G, Jenuwein T (2006) Jmjd2b antagonizes H3K9 trimethylation at pericentric heterochromatin in mammalian cells. *Genes Dev* **20:** 1557-1562

Fujita N, Watanabe S, Ichimura T, Tsuruzoe S, Shinkai Y, Tachibana M, Chiba T, Nakao M (2003) Methyl-CpG Binding Domain 1 (MBD1) Interacts with the Suv39h1-HP1 Heterochromatic Complex for DNA Methylation-based Transcriptional Repression. *J Biol Chem* **278:** 24132-24138

Fuks F, Hurd PJ, Deplus R, Kouzarides T (2003) The DNA methyltransferases associate with HP1 and the SUV39H1 histone methyltransferase. *Nucleic Acids Res* **31:** 2305-2312

Garcia De La Torre J, Huertas ML, Carrasco B (2000) Calculation of hydrodynamic properties of globular proteins from their atomic-level structure. *Biophys J* **78:** 719-730

Gillespie DT (2007) Stochastic simulation of chemical kinetics. *Annu Rev Phys Chem* **58:** 35-55

Hahn M, Dambacher S, Dulev S, Kuznetsova AY, Eck S, Worz S, Sadic D, Schulte M, Mallm JP, Maiser A, Debs P, von Melchner H, Leonhardt H, Schermelleh L, Rohr K, Rippe K, Storchova Z, Schotta G (2013) Suv4-20h2 mediates chromatin compaction and is important for cohesin recruitment to heterochromatin. *Genes Dev* **27:** 859-872

Hendrich B, Bird A (1998) Identification and characterization of a family of mammalian methyl-CpG binding proteins. *Mol Cell Biol* **18:** 6538-6547

Jegou T, Chung I, Heuvelmann G, Wachsmuth M, Görisch SM, Greulich-Bode K, Boukamp P, Lichter P, Rippe K (2009) Dynamics of telomeres and promyelocytic leukemia nuclear bodies in a telomerase negative human cell line. *Mol Biol Cell* **20:** 2070-2082

Kepert JF, Fejes Tóth K, Caudron M, Mücke N, Langowski J, Rippe K (2003) Conformation of reconstituted mononucleosomes and effect of linker histone H1 binding studied by scanning force microscopy. *Biophys J* **85:** 4012-4022

Kimura H, Shiota K (2003) Methyl-CpG-binding protein, MeCP2, is a target molecule for maintenance DNA methyltransferase, Dnmt1. *J Biol Chem* **278:** 4806-4812

Laue TM, Shah BD, Ridgeway TM, Pelletier SL (1992) Computer aided interpretation of analytical sedimentation data for proteins. In *Analytical Ultracentrifugation in Biochemistry and Polymer Science*, Harding SE, Rowe AJ, Horton JC (eds), pp 90-125. Cambridge: Royal Society of Chemistry

Lehnertz B, Ueda Y, Derijck AA, Braunschweig U, Perez-Burgos L, Kubicek S, Chen T, Li E, Jenuwein T, Peters AH (2003) Suv39h-mediated histone H3 lysine 9 methylation directs DNA methylation to major satellite repeats at pericentric heterochromatin. *Curr Biol* **13:** 1192-1200

Lunyak VV, Burgess R, Prefontaine GG, Nelson C, Sze SH, Chenoweth J, Schwartz P, Pevzner PA, Glass C, Mandel G, Rosenfeld MG (2002) Corepressor-dependent silencing of chromosomal regions encoding neuronal genes. *Science* **298:** 1747-1752

Maison C, Bailly D, Peters AH, Quivy JP, Roche D, Taddei A, Lachner M, Jenuwein T, Almouzni G (2002) Higher-order structure in pericentric heterochromatin involves a distinct pattern of histone modification and an RNA component. *Nat Genet* **30:** 329-334

Muller CB, Weiss K, Richtering W, Loman A, Enderlein J (2008) Calibrating differential interference contrast microscopy with dual-focus fluorescence correlation spectroscopy. *Opt Express* **16:** 4322-4329

Müller KP, Erdel F, Caudron-Herger M, Marth C, Fodor BD, Richter M, Scaranaro M, Beaudouin J, Wachsmuth M, Rippe K (2009) Multiscale analysis of dynamics and interactions of heterochromatin protein 1 by fluorescence fluctuation microscopy. *Biophys J* **97:** 2876-2885

Pack C, Saito K, Tamura M, Kinjo M (2006) Microenvironment and effect of energy depletion in the nucleus analyzed by mobility of multiple oligomeric EGFPs. *Biophys J* **91:** 3921-3936

Peters AH, O'Carroll D, Scherthan H, Mechtler K, Sauer S, Schofer C, Weipoltshammer K, Pagani M, Lachner M, Kohlmaier A, Opravil S, Doyle M, Sibilia M, Jenuwein T (2001) Loss of the Suv39h histone methyltransferases impairs mammalian heterochromatin and genome stability. *Cell* **107:** 323-337

Phillip Y, Kiss V, Schreiber G (2012) Protein-binding dynamics imaged in a living cell. *Proc Natl Acad Sci USA* **109:** 1461-1466

Philo JS (2000) A method for directly fitting the time derivative of sedimentation velocity data and an alternative algorithm for calculating sedimentation coefficient distribution functions. *Anal Biochem* **279:** 151-163

Rea S, Eisenhaber F, O'Carroll D, Strahl BD, Sun ZW, Schmid M, Opravil S, Mechtler K, Ponting CP, Allis CD, Jenuwein T (2000) Regulation of chromatin structure by site-specific histone H3 methyltransferases. *Nature* **406:** 593-599

Rippe K (2001) Making contacts on a nucleic acid polymer. *Trends Biochem Sci* **26:** 733-740

Rippe K, von Hippel PH, Langowski J (1995) Action at a distance: DNA-looping and initiation of transcription. *Trends Biochem Sci* **20:** 500-506

Rothbauer U, Zolghadr K, Muyldermans S, Schepers A, Cardoso MC, Leonhardt H (2008) A versatile nanotrap for biochemical and functional studies with fluorescent fusion proteins. *Mol Cell Proteomics* **7:** 282-289

Rothbauer U, Zolghadr K, Tillib S, Nowak D, Schermelleh L, Gahl A, Backmann N, Conrath K, Muyldermans S, Cardoso MC, Leonhardt H (2006) Targeting and tracing antigens in live cells with fluorescent nanobodies. *Nat Meth* **3:** 887-889

Schotta G, Lachner M, Sarma K, Ebert A, Sengupta R, Reuter G, Reinberg D, Jenuwein T (2004) A silencing pathway to induce H3-K9 and H4-K20 trimethylation at constitutive heterochromatin. *Genes Dev* **18:** 1251-1262

Schotta G, Sengupta R, Kubicek S, Malin S, Kauer M, Callen E, Celeste A, Pagani M, Opravil S, De La Rosa-Velazquez IA, Espejo A, Bedford M, Nussenzweig A, Busslinger M, Jenuwein T (2008) A chromatin-wide transition to H4K20 mono-methylation impairs genome integrity and programmed DNA rearrangements in the mouse. *Genes Dev* **22:** 2048-2061

Schuck P (2003) On the analysis of protein self-association by sedimentation velocity analytical ultracentrifugation. *Anal Biochem* **320:** 104-124

Smallwood A, Esteve PO, Pradhan S, Carey M (2007) Functional cooperation between HP1 and DNMT1 mediates gene silencing. *Genes Dev* **21:** 1169-1178

Sprague BL, Pego RL, Stavreva DA, McNally JG (2004) Analysis of binding reactions by fluorescence recovery after photobleaching. *Biophys J* **86:** 3473-3495

Taddei A, Maison C, Roche D, Almouzni G (2001) Reversible disruption of pericentric heterochromatin and centromere function by inhibiting deacetylases. *Nat Cell Biol* **3:** 114-120

Teif VB, Vainshtein Y, Caudron-Herger M, Mallm JP, Marth C, Hofer T, Rippe K (2012) Genome-wide nucleosome positioning during embryonic stem cell development. *Nat Struct Mol Biol* **19:** 1185-1191

Wachsmuth M, Caudron-Herger M, Rippe K (2008) Genome organization: Balancing stability and plasticity. *Biochim Biophys Acta* **1783:** 2061-2079

Waterston RH, Lindblad-Toh K, Birney E, Rogers J, Abril JF, Agarwal P, Agarwala R, Ainscough R, Alexandersson M, An P, Antonarakis SE, Attwood J, Baertsch R, Bailey J, Barlow K, Beck S, Berry E, Birren B, Bloom T, Bork P et al (2002) Initial sequencing and comparative analysis of the mouse genome. *Nature* **420:** 520-562

Weidemann T, Wachsmuth M, Tewes M, Rippe K, Langowski J (2002) Analysis of ligand binding by two-colour fluorescence cross-correlation spectroscopy. *Single Mol* **3:** 49-61

Zee BM, Levin RS, Xu B, LeRoy G, Wingreen NS, Garcia BA (2010) In vivo residue-specific histone methylation dynamics. *J Biol Chem* **285:** 3341-3350

Zolghadr K, Mortusewicz O, Rothbauer U, Kleinhans R, Goehler H, Wanker EE, Cardoso MC, Leonhardt H (2008) A fluorescent two-hybrid assay for direct visualization of protein interactions in living cells. *Mol Cell Proteomics* **7:** 2279-2287
